# Evaluating the Impact of High- and Low-Starch Diets on the Ruminal Microbiota of Dairy Cows

**DOI:** 10.3390/microorganisms14091968

**Published:** 2026-09-06

**Authors:** Dino L. Sbardellati, Amelie Fischer, Madison S. Cox, Wenli Li, Kenneth F. Kalscheur, Garret Suen

**Affiliations:** 1 Department of Bacteriology, University of Wisconsin-Madison, Madison, WI 53706, USA; dino.sbardellati@cuanschutz.edu (D.L.S.); maddie.shay.cox@gmail.com (M.S.C.); 2 Oak Ridge Institute for Science and Education, Oak Ridge, TN 37830, USA; amelie.fischer67@gmail.com; 3 US Dairy Forage Research Center, USDA-Agricultural Research Service, Madison, WI 53706, USA; wenli.li@ars.usda.gov (W.L.); kenneth.kalscheur@usda.gov (K.F.K.); 4 Microbiology Doctoral Training Program, University of Wisconsin-Madison, Madison, WI 53706, USA; 5 Division of Allergy and Infectious Diseases, Department of Medicine, University of Washington, Seattle, WA 98109, USA

**Keywords:** 16S rRNA, dairy cows, diet, epimural, microbiota, rumen

## Abstract

Ruminants harbor a ruminal microbiome that converts their host-indigestible diet into nutrients. This microbiome contains three distinct communities: liquid, solid, and epithelial (or epimural). Currently, there is limited research examining the diet-dependent responses of all three microbiomes within the same animal. Here, we used next-generation 16S rRNA sequencing to characterize the ruminal solid (RS), liquid (RL), and epimural (RE) microbiotas of 13 lactating and cannulated Holstein dairy cows fed either a high- or low-starch diet in a crossover experimental design. Independent of diet, we found that all sample types were dominated by the phyla Firmicutes and Bacteroidetes. Proteobacteria and Epsilonbacteraeota were also highly abundant but only in the RE. Although the total VFA molar abundance did not differ between diets, propionate and valerate were found to increase with the high-starch diet, while acetate was increased with the low-starch diet. Overall, we found that the RE microbiota was more diverse than the RS and RL communities, with diet impacting community diversity in the RS. We found that sample type, diet treatment, and their interaction significantly impacted community structure and composition. Notably, *Prevotella* was most abundant in the RL and RS, particularly on the low-starch diet. We also found that Lachnospiraceae were significantly enriched in the RS and RL with a high-starch diet, whereas *Succiniclasticum* was highly abundant in the RE with a low-starch diet. These data suggest that diet influences all three ruminal microbiomes and provides a useful framework for understanding the role of these microbiomes in mediating host production.

## 1. Introduction

The ability of bovines to produce animal products from plant matter is facilitated by their unique biology, specifically their rumen and its associated microbiome [1,2,3]. Ruminal microbes possess metabolic capabilities not present in their ruminant host and are able to convert host-indigestible dietary components into nutrients that can be used by the host [1,4]. Volatile fatty acids (VFAs) produced by rumen microbial fermentation are absorbed by the host across the rumen epithelium and comprise >70% of the ruminant’s energetic requirements [5].

The rumen microbiome consists of three distinct microbiotas: the ruminal solid, ruminal liquid, and epithelial (or epimural) communities [1,3,6]. Each of these communities has been shown to be distinct, and they are hypothesized to perform different functions within the rumen [3,7]. Ruminal solid bacteria can be found adhered to feed particles and plant fibers and represent ~70% of the total bacterial biomass in the rumen [3,4]. This community is mainly composed of bacteria in the phyla Firmicutes and Fibrobacteres and are composed of cellulolytic organisms such as *Fibrobacter succinogenes* and *Ruminococcus* spp., which are responsible for the degradation of recalcitrant carbohydrates in plant cell walls [4]. In addition, members of the Bacteroides such as the *Prevotella* spp. are also present and serve to synergistically facilitate the breakdown of plant cellulose and hemicellulose fibers for the rest of the ruminal microbiome.

In contrast, ruminal liquid bacteria are found floating freely within the liquid phase of the rumen and comprise ~30% of the total bacterial biomass in the rumen [3,4]. This community is typically composed of saccharolytic and proteolytic microbes, such as *Prevotella* spp., *Butyrivibrio* spp., and *Selenomonas* spp., which are responsible for converting solubilized substrates into VFAs such as acetate, butyrate, and propionate, which serve as the primary energy source for the host [4]. In addition, the liquid microbiota also serves as a major source of microbial protein for the host.

The epimural bacteria are found associated with the epithelial tissue of the rumen and comprise the remainder (<1%) of the total bacterial biomass in the rumen [3,4]. Relative to the solid and liquid microbiota, this bacterial community is characterized by a higher abundance of Proteobacteria [8] and specialist bacteria in the genera *Campylobacter* and *Mogibacterium* [9]. Despite their comparatively low numbers, the epimural bacterial community is hypothesized to play many important roles due to its spatial placement at the interface between the host and the luminal microbiota [2,10]. These include oxygen scavenging, nitrogen cycling, and the degradation of sloughed host epithelial tissue [10,11,12,13]. Moreover, these bacteria likely also serve as key conduits for the absorption of VFAs across the ruminal epithelium given their presence at the rumen–microbial interface.

Although some studies have explored the epimural community [7,8], little is known regarding how it is impacted by host diet. This is likely due to the difficulty associated with sampling the epimural microbiota, which typically requires sampling from cannulated animals. As such, only a handful of studies have explored the epimural microbiota from the perspective of dietary additives or ruminal disorders [14,15]. To address this gap in knowledge, we used next-generation 16S rRNA sequencing to characterize the bacterial communities associated with the ruminal solid (RS), liquid (RL), and epimural (RE) microbiotas of 13 lactating and cannulated Holstein dairy cows fed either a high- or low-starch diet in a crossover experimental design. We hypothesized that the microbiota associated with the RS, RL, and RE communities will be different and that changes in host diet will lead to significant changes in each of these individual microbiotas.

## 2. Materials and Methods

### 2.1. Diet and Experimental Design

Cows used in this study were a subset of the animals used in a previous study by Fischer et al. [16]. Briefly, two cohorts were assigned from an initial population of 62 cows (29 primiparous and 33 multiparous cows) at the US Dairy Forage Research Center farm (USDFRC, Prairie du Sac, WI, USA) based on parity, dry matter intake (DMI), milk net energy, body weight (BW), body conditional score, BW loss and BW gain, as assessed over a 31-day pre-experimental period (T_0_) where animals were fed a common diet. The average days in milk (DIM) was 136 (±23) d for the first cohort and 138 (±23) d for the second cohort at the start of the experimental period. From these two cohorts, 13 were randomly selected for cannulation and subjected to a dietary crossover experimental design with animals fed either a high- or low-starch diet (Supplementary Table S1) across two sampling periods. Group one (*n* = 6) started on the high-starch diet and ended on the low-starch diet. Conversely, group two (n = 7) started on the low-starch diet and ended on the high-starch diet. Within each experimental period, the first 2 weeks were used for diet transition, and the remaining 8 weeks were used for the experiment. Cows were allowed ad libitum access to water and feed throughout the experiment.

Both diets were defined according to their starch content, with the high-starch diet containing ~27% dry matter as starch and the low-starch diet containing ~13% of dry matter as starch. Dietary formulations and their composition values are described in Fischer et al. [16] and were determined from once-a-day sampling of forages and once-a-week sampling of concentrate. Briefly, samples were initially dried for 48 h at 55 °C and were subsequently dried for 24 h at 105 °C. For each experimental period, diets were composited on a dry matter basis into 4-week samples and analyzed for nutritional composition by Dairyland Laboratories Inc. (Arcadia, WI, USA) as follows: ash (method 942.05), crude protein (method 990.03), neutral detergent fiber (method 2002.04), acid detergent fiber (method 973.18), lignin (method 973.18), ether extract (method 920.39), and starch (method 996.11).

Ruminal contents and epithelial samples for bacterial community sequencing were collected after the morning feeding on the last day of each 8-week period, prior to diet transition to the next period. All animal work described in this study was performed in accordance with approved guidelines and regulations by The University of Wisconsin-Madison’s Institutional Animal Care and Use Committee under protocol #A005945 (date 9 January 2018).

### 2.2. Ruminal Volatile Fatty Acid Quantification

Volatile fatty acids (VFAs) were measured from ruminal samples collected 2 h prior to morning feeding and 2 weeks prior to the end of each diet treatment period using high-performance liquid chromatography (HPLC) on a Bio-Rad HPX-87H instrument with a 300 mm length column and a 4.6 mm i.d (Bio-Rad, Bio-Rad Laboratories, Hercules, CA, USA), according to the method established by Weimer et al. [17]. Volatile fatty acids were identified by comparison of retention time with a mixture of fermentation product standards (10 mM each), as previously described [18].

### 2.3. Rumen Epithelial Sampling

Ventral sac rumen wall epithelium (RE) samples were collected at the end of each 8-week diet treatment period. Sampling took place 3 to 4 h after the morning feeding. Rumen evacuations and epithelial sampling took about 25 min. During this time, cows had no access to feed or water. Epithelial samples were collected using Jackson Uterine Biopsy Forceps (Jorgensen Laboratories, Loveland, CO, USA) following complete removal of the rumen contents via the rumen cannula. Immediately after tissue sampling, rumen contents were placed back into the rumen (within 30 min of removal) and access to water and feed were restored. Immediately following collection, tissue samples were washed in sterile 1× PBS and snap frozen in liquid nitrogen. Epithelial samples were transferred and stored at −80 °C until DNA extraction.

### 2.4. Rumen Content Sampling

Total rumen content samples from each animal were collected via the rumen cannula at the end of each 8-week diet treatment period. Sampling took place 3 to 4 h after the morning feeding, concomitant with rumen epimural sampling. Bulk rumen contents were then frozen at −80 °C pending further processing. Total contents were thawed overnight at 4 °C, and rumen liquid (RL) was collected in a 50 mL Falcon conical tube (Corning, Glendale, AZ, USA) by straining a subsample of the total rumen contents through four layers of cheesecloth. The remaining rumen solid (RS) material was further squeezed to remove liquid and transferred into a separate 50 mL conical tube. All samples were immediately transported on wet ice and stored at −80 °C prior to DNA extraction.

### 2.5. DNA Extraction

Total genomic DNA was extracted from the rumen solids and liquids following a mechanical disruption and phenol–chloroform extraction protocol adapted from Dill-Mcfarland et al. [19]. In short, roughly 10 mL of rumen solid material were combined with 40 mL of chilled DNA extraction buffer [100 mM Tris-HCl, 10 mM EDTA, 0.15 M NaCl (pH 8.0)] and homogenized for 5 min in a stomacher bag with 0.5 mm filter lining using a Tekmar STO-400 paddle stomacher (Seward Inc., Bohemia, NY, USA). The resulting homogenate was then transferred to a new sterile 50 mL Falcon conical tube and spun, along with thawed rumen fluid samples, at 5000 g for 1 h to pellet the cells. This supernatant was then removed, and ~10 mL of cold DNA extraction buffer was added. The samples were then vortexed until homogenized using a GeneMate Vortex Mixer (BioExpress, Kaysville, UT, USA). A total of 1 mL of the resulting re-homogenized samples was then transferred to a bead-beat tube containing 0.5 g of 0.1 mm silica beads, combined with 50 uL of 20% SDS and 700 uL of cold equilibrated phenol (pH 8.0), and then bead-beat for 2 min in a Mini-Beadbeater 96 (BioSpec Products Inc., Bartlesville, OK, USA). The samples were then incubated at 60 °C and subjected to another round of bead beating. Aqueous-phase crude extracts were then washed twice with cold phenol:chloroform:isoamyl alcohol (25:24:1) and precipitated overnight at −20 °C with 2 M sodium acetate and isopropanol. Cell pellets were washed with cold ethanol, dried, and suspended in 20 μL of Tris-EDTA buffer. DNA was quantified using a Qubit Fluorometer (Invitrogen, San Diego, CA, USA) and stored at −80 °C until library preparation.

Ruminal wall tissue samples were processed for total DNA extraction using a protocol adapted from Roussel et al. [20] and described in Sbardellati et al. [8]. In short, 0.25 to 0.5 g of epithelial tissue was subsampled from biopsies using sterile tissue shears and combined with 1 mL of chilled DNA extraction buffer [100 mM Tris-HCl, 10 mM EDTA, 0.15 M NaCl (pH 8.0)] and a 4 mm silica bead (BioSpec Products Inc., Bartlesville, OK, USA). The samples were vortexed on high 5 times (30 s each time) using a GeneMate Vortex Mixer (BioExpress, Kaysville, UT, USA). The resulting supernatants were combined with phenol and 20% SDS and mechanically disrupted using 0.1 mm silica beads (BioSpec Products Inc.) and a Mini-Beadbeater 96 (BioSpec Products Inc.). The samples were then incubated at 60 °C and subjected to another round of bead beating. Aqueous-phase crude extracts were then washed twice with cold phenol:chloroform:isoamyl alcohol (25:24:1) and precipitated overnight at −20 °C with 2 M sodium acetate and isopropanol. Cell pellets were washed with cold ethanol, dried, and suspended in 20 μL of Tris-EDTA buffer. DNA was quantified using a Qubit Fluorometer and stored at −80 °C until library preparation.

### 2.6. PCR and Sequencing

PCR was preformed using universal bacterial primers (515F: 5′-GTGCCAGCMGCCGCGGTAA-3′; 806R: 5′-GGACTACHVGGGTWTCTAAT-3′) targeting the variable 4 (V4) region of the 16S rRNA gene, as described by Kozich et al. [21]. These primers also included adapters suitable for sequencing using the Illumina (Illumina Inc., San Diego, CA, USA) sequencing system (F: 5′-AATGATACGGCGACCACCGAGATCTACAC-3′; R: 5′-CAAGCAGAAGACGGCATACGAGAT-3′). To facilitate multiplexing, unique barcodes were also included as follows: the forward primers had 16 unique 8 bp barcodes, whereas the reverse primers had 24 unique 8 bp barcodes. The complete set of primers used for PCR is provided in Supplementary Table S2. PCRs were performed with the following composition: 10 ng of template DNA, 0.5 μL of forward primer, 0.5 μL of reverse primer, 12.5 μL of KAPA 2× HiFi Master-mix (KAPA Biosystems, Wilmington, MA, USA), and water to 25 μL. The cycle conditions were as follows: an initial denaturing step of 3 min at 95 °C; followed by 25 cycles of 30 s at 95 °C, 30 s at 55 °C, and 30 s at 72 °C; and a final elongation step of 5 min at 72 °C. The subsequent PCR products were purified via gel extraction from 1% low-melt agarose (National Diagnostics, Atlanta, GA, USA) using a ZR-96 Zymoclean Gel DNA Recovery Kit (Zymo Research, Irvine, CA, USA). Bands of approximately 350 bp, along with extraction and PCR negatives, were collected and processed according to the manufacturer’s description. The purified PCR products were quantified using a Qubit Fluorometer and equimolarly pooled into a single library. This library was then diluted to 4 nM and sequenced using a MiSeq 2 × 250 v2 kit (Illumina Inc., San Diego, CA, USA) on an Illumina MiSeq sequencer (Illumina Inc., San Diego, CA, USA) using custom primers (Read 1: 5′- TATGGTAATTGTGTGCCAGCMGCCGCGGTAA-3′; Read 2: 5′-AGTCAGTCAGCCGGACTACHVGGGTWTCTAAT-3′) [21]. The raw sequence data were deposited into the National Center for Biotechnological Information’s Short Read Archive and are publicly available under accession number PRJNA562284.

### 2.7. Bioinformatic Sequence Clean-Up and Classification

Raw sequences were demultiplexed on the Illumina MiSeq, and the resulting fastq files were processed in mothur v1.41.2 [22] following a protocol adapted from Schloss et al. [22]. Briefly, the function *make.contigs* was used to assemble single contiguous regions (contigs) from the raw, paired end and overlapping sequencing reads. These resulting contigs were screened using the function *screen.seqs* to remove contigs with ambiguous base pairs, contigs >300 bp, or contigs with homopolymers >8. The remaining reads were aligned against the SILVA 16S rRNA database version 138.2 [23] with a minimum 80% confidence assignment. Any sequence not aligned to the V4 region was subsequently dropped. Amplicon sequences with a maximum difference of n = 2 were then pooled using pre.cluster. Chimeras were identified using the function chimera.uchime and removed with remove. seqs. Next, the function *remove.lineage* was used to remove nonbacterial sequences. Taxonomy was assigned via *classify.otu* using the mothur-formatted SILVA database v138.2 (https://mothur.org/wiki/Silva_reference_files (accessed on 9 July 2025)). Sequences were then binned into operational taxonomic units (OTUs) at 97% similarity using the function *cluster.split* with the opticlust method. Finally, the function *normalize.shared* was used to normalize the number of sequences per sample to the lowest number of sequences observed in a non-negative sample. Both the sequence coverage, assessed as Good’s coverage [24], and α-diversity, measured as Shannon’s diversity index [25] and Chao’s species richness [26], were calculated in mothur using *summary.single*.

### 2.8. Statistical Analysis

Statistical tests and analyses were performed in R (v3.6.1, https://www.r-project.org/; R Core Team, 2019). The normality of each VFA molar proportion, Shannon’s diversity, and Chao’s richness distributions were assessed both qualitatively, via inspection of quantile–quantile plots, and quantitatively, via Shapiro–Wilk (*shapiro.test*) tests. The homogeneity of variance of each of these distributions was then assessed using Levene’s test (leveneTest) with the median serving as the data’s center. A combination of hypothesis tests, both T (*t.test*) and Wilcoxon (*wilcox.test*) tests, and linear mixed-effect models (lme4::lmer) [27] were used to explore whether diet order (the interaction of diet and time) significantly impacted the VFA molar proportion, Shannon’s diversity, and Chao’s richness distributions. The significance of changes in VFAs in response to changes in diet was assessed using either paired *t*-tests or paired Wilcoxon tests, depending on the type of distribution. Similarly, the response of the Shannon and Chao α-diversity metrics within individual sample types to changes in diet were measured using paired hypothesis tests (either *t*-tests or paired Wilcoxon tests). To measure overall differences in Shannon’s diversity and Chao’s richness between sampling locations (RL, RS, and RE) on the same diet, analysis of variance (*aov*) followed by Tukey’s honestly significant difference (TukeyHSD) tests were used.

For β-diversity, both the Bray–Curtis (community structure) and Jaccard (community composition) metrics were calculated and plotted using the vegan::metaMDS function (vegan package) [28] in R. Standard error ellipses were plotted to visualize the center and variability of the different groups’ point distributions. Differences in the variability of those groups were assessed by comparing each point distribution’s spread via the beta-dispersion test vegan::betadisper. Permutational ANOVA (PERMANOVA) tests were then used to assess the significance of each groups’ separation. If the variability of all tested groups was comparable, the parametric adonis test were used. If the variability of the tested groups was not comparable, non-parametric analysis of similarity (ANOSIM) tests were used.

To determine if the order in which animals received the diets had a significant effect on β-diversity, two different approaches were used. The first approach used PERMANOVA tests to compare the significance of separation of each diet-order group’s β-diversity point distribution (i.e., RE_high-starch_, time 1 compared to RE_high-starch_, time 2). The second approach calculated pairwise Bray–Curtis and Jaccard dissimilarity comparisons for specific sample types, taken from the same animals but on different diets (i.e., RL_cow1_high-starch_ vs. RL_cow1_low-starch_), for all animals in this study. This second approach resulted in two dissimilarity distributions for each sample type, corresponding to the two diet-order groups (i.e., RL_high-starch→low-starch_ vs. RL_low-starch→high-starch_). These two distributions were then tested for significant differences using standard hypothesis tests. If no significant differences were observed between individual sample type diet-order distributions, then the order in which animals received diets was deemed to not impact β-diversity. If diet order was concluded to not impact β-diversity, the two diet-order dissimilarity distributions for each sample type were pooled, and overall differences between each pairwise sample type’s response to diet were compared using ANOVA and TukeyHSD tests.

Vegan::simper was used to explore which variables (bacterial genera) were the most important in distinguishing the bacterial communities associated with different rumen sample types, diets, and the interaction of these factors. The total SIMPER output provided by R was subset to retain only those genera that accumulatively explained 90% of the dissimilarity and that SIMPER identified as differing significantly between grouping factors.

Differences in sequence abundance at different taxonomic resolutions were assessed using Kruskal–Wallis (Kruskal.test) and pairwise-Wilcoxon (pairwise.wilcox.test) tests with FDR *p*-value corrections. Venn diagrams were constructed using only those OTUs present at >0.1% relative abundance within a single sample.

## 3. Results

### 3.1. Sequencing Results

Ruminal liquid (RE), solid (RS), and epithelial (RE) samples were collected from 13 different animals at two different timepoints, yielding a total of 78 samples. After filtering in mothur, these samples generated a total of 3,818,831 high-quality 16S rRNA bacterial sequences. Sequence coverage was deemed sufficient, with Good’s coverage values >95% for all bacterial community samples. Samples were then normalized to 9720 sequences per sample, corresponding to the number of sequences within the sample containing the lowest number of observed sequences in the data set.

### 3.2. Total VFAs Do Not Differ by Diet but by the Molar Proportion of Individual VFAs

**Figure 1:**
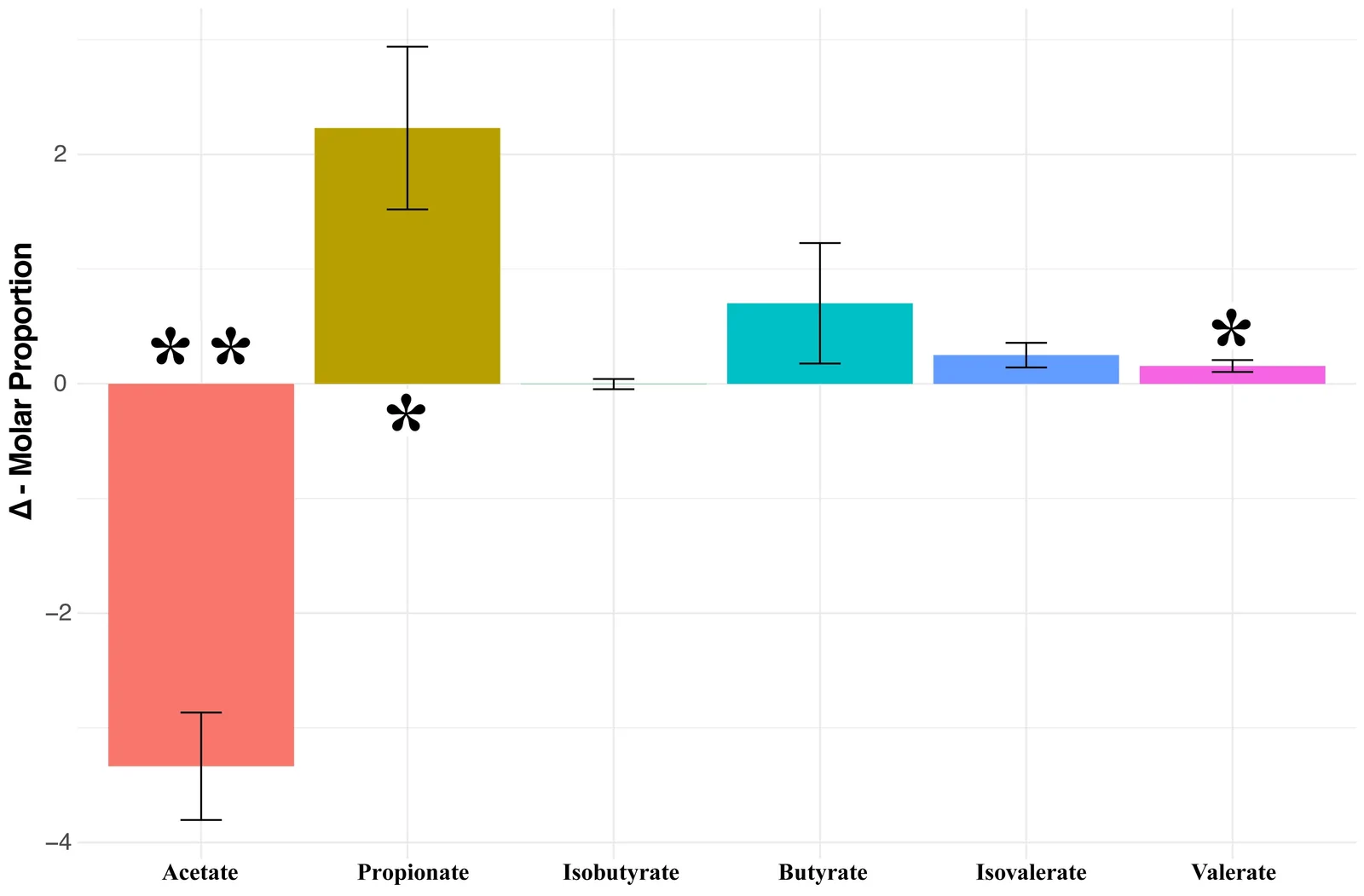
Bar plot displaying the change in molar proportion of different volatile fatty acids (VFAs) in response to diet. Bars represent the distributions obtained by taking each animal’s VFA molar proportion under high-starch diet and subtracting that animal’s VFA molar proportion under low-starch diet. Asterisks represent the level of significance obtained by testing these results with either paired *t*-tests or non-parametric paired Wilcoxon tests (* *p* ≤ 0.01; ** *p* ≤ 0.001).

Of all the VFAs measured in this study, only the branched-chain VFAs (isobutyrate and isovalerate) were impacted by diet order (isobutyrate—lmer *p*-value < 0.001; isovalerate—t.test *p*-value < 0.05). We found that, while the molar proportions of individual VFAs differed significantly, the total molar concentration of all VFAs did not (Figure 1 and Supplementary Table S3). Specifically, the molar proportion of acetate was found to be higher in the low-starch diet (*p* < 0.001), while the molar proportions of propionate and valerate were both higher in the high-starch diet (propionate *p* < 0.01; valerate *p* < 0.05). The molar proportion of isovalerate was found to be higher in the high-starch diet (*p* < 0.05) but only for the low-starch to high-starch diet-order group. Butyrate and isobutyrate (both diet-order groups) did not differ significantly by diet.

### 3.3. The Epimural Microbiota Is More Diverse, Rich, and Stable Relative to Luminal Communities

**Figure 2:**
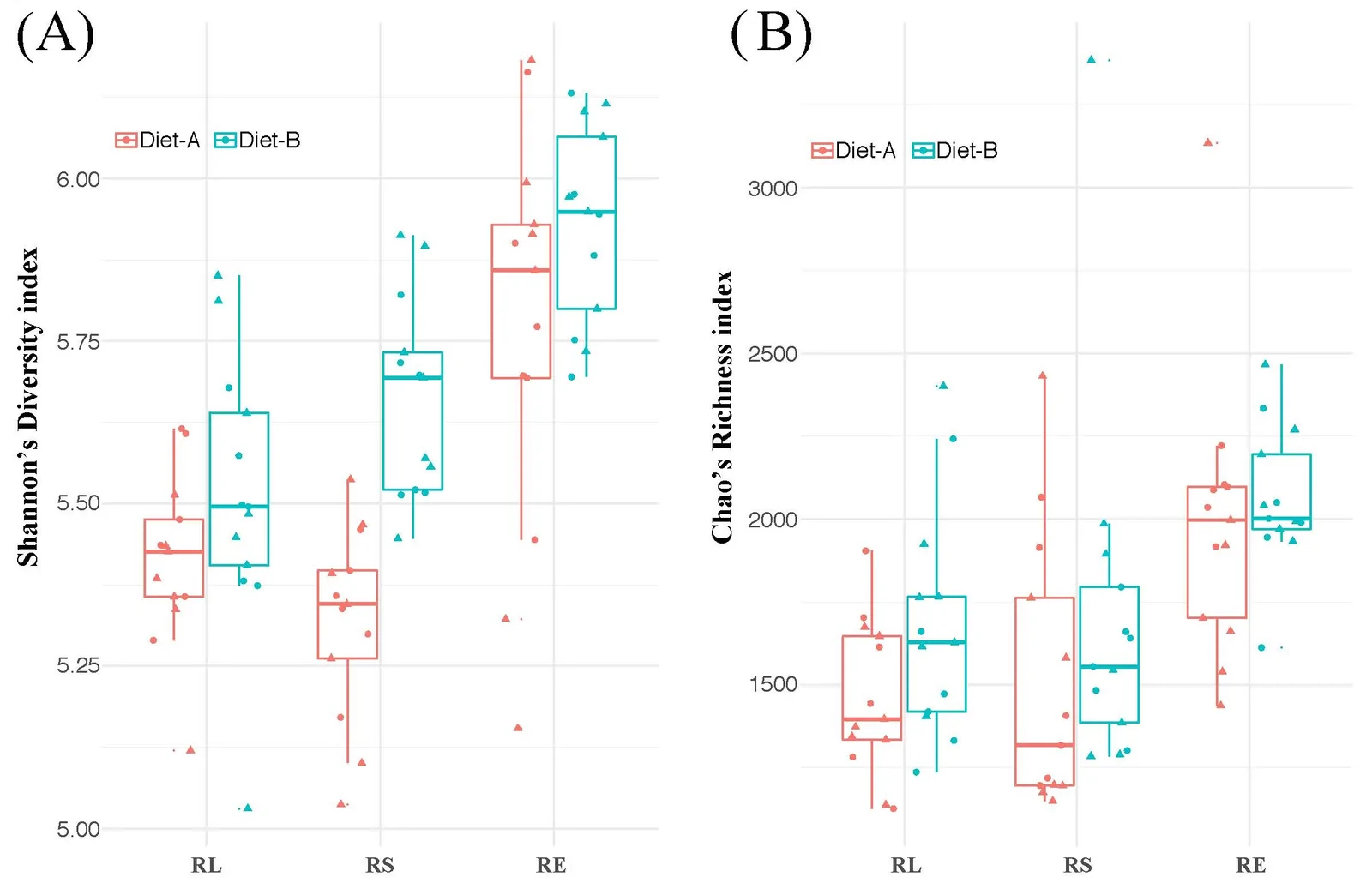
Box plots presenting (**A**) Shannon’s diversity and (**B**) Chao’s richness of the rumen liquid (RL), rumen solid (RS), and rumen epimural (RE) bacterial communities with each diet. Diet-A = high-starch; Diet-B = low-starch.

Our analysis of α-diversity (Shannon’s diversity or Chao’s richness) showed that it was not significantly impacted by diet order, regardless of sample type. Comparisons of α-diversity between different sample types (Figure 2) revealed that, independent of diet, the RE communities were consistently higher in Shannon’s diversity, when compared to either of the luminal (RL and RS) microbial communities (all *p* < 0.001). The same was observed for Chao’s richness (all *p* < 0.05) with the exception of the low-starch RS-RE comparison, which only tended (*p* = 0.072) to differ. The luminal microbial communities (RL and RS) did not differ from one another in either α-diversity metric regardless of diet. Within individual sample types (Figure 2), we found that only the Shannon’s diversity of the RS was impacted by diet, with the low-starch diet resulting in a higher Shannon’s diversity score (*p* < 0.001).

### 3.4. Diet and Sample Type Significantly Impact Bacterial Community Structure and Composition

**Figure 3:**
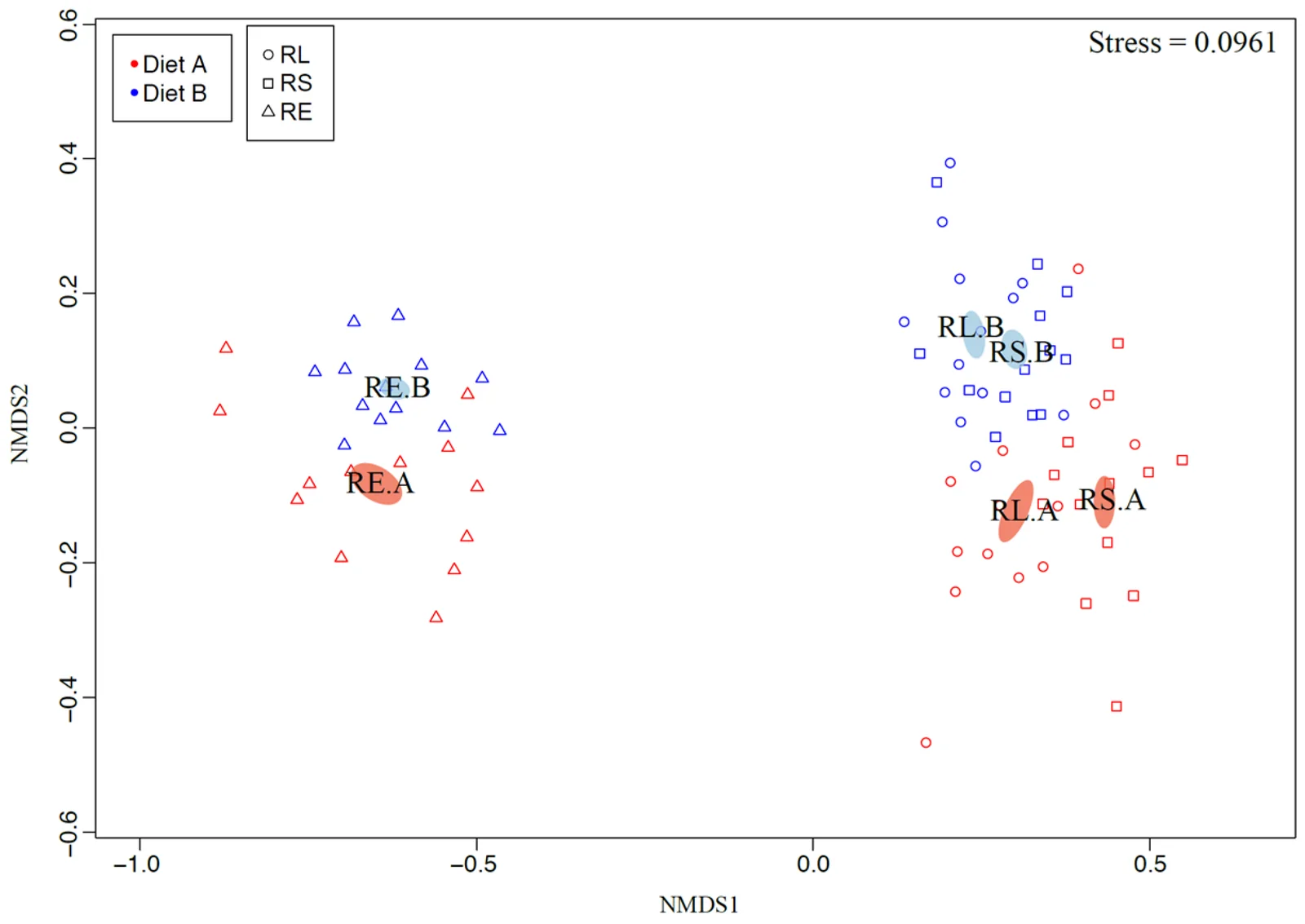
Two-dimensional, non-metric multidimensional scaling (nMDS) plot displaying the Bray–Curtis β-diversity of rumen liquid (RL)-, rumen solid (RS)-, and rumen epimural (RE)-associated bacterial communities. Each point represents a single sample, and the distance between points represents the dissimilarity between each sample’s bacterial communities. Standard error ellipses show the center and variability of each point distribution. Samples are color coordinated by diet, where red (Diet A) corresponds to the high-starch diet and blue (Diet B) to the low-starch diet. Stress = 0.0961.

**Figure 4:**
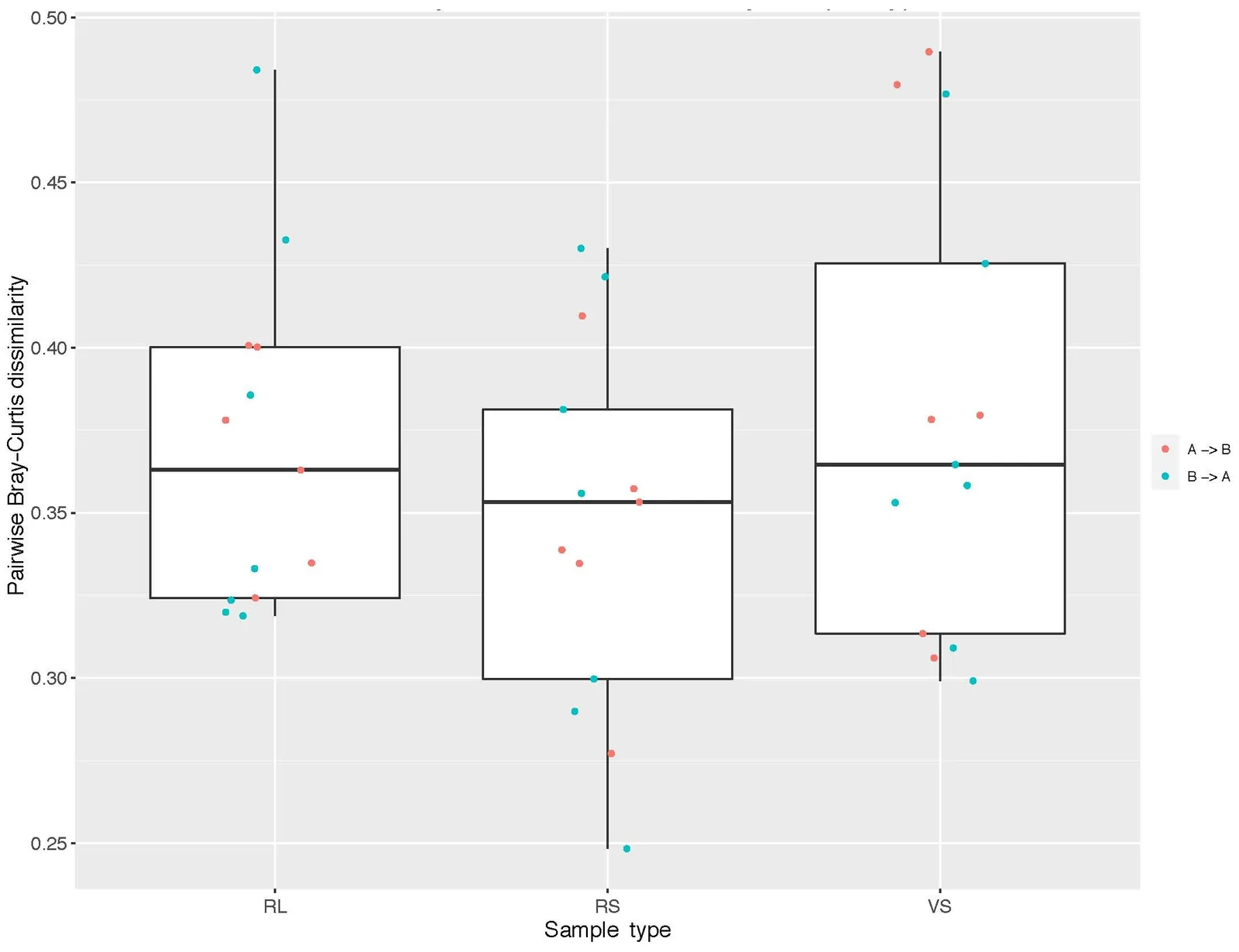
Box plots of pairwise Bray–Curtis dissimilarity distributions representing the overall change in rumen liquid (RL), rumen solid (RS) and rumen epimural (RE) communities in response to diet. Dissimilarity distributions were generated by calculating the pairwise Bray–Curtis dissimilarity of specific sample types taken from the same animal but on different diets (i.e., RLcow1_high-starch vs. RLcow1_low-starch) and represent the overall “linear” distance between paired samples in β-diversity space. Each point represents either the transition from a high-starch (A) to a low-starch (B) diet in red or vice versa in blue.

Diet order did not significantly impact either the Bray–Curtis (community structure) or Jaccard (community composition) β-diversity metrics, although there was clear separation of samples based on type, diet, and the interaction of these factors (Figure 3). Within individual diets, all separations due to sample type were found to be significant (RS-RL high-starch *p* < 0.01; all others *p* < 0.001). Additionally, within sample types, all separations due to diet were also found to be significant (all *p* < 0.001). With regard to the RE samples, the microbial community with the low-starch diet was found to be significantly less varied, in terms of both the Bray–Curtis and Jaccard metrics (*p*< 0.001). Finally, β-diversity pairwise-comparisons of different sample types, derived from the same animals fed different diets (Figure 4), suggest that the “linear” distance between these paired samples is equal for all sample types and that, overall, all sample types change to a similar degree in response to changes in diet.

### 3.5. A High-Starch Diet Results in a Greater Number of Unique RE OTUs Relative to a Low-Starch Diet

**Figure 5:**
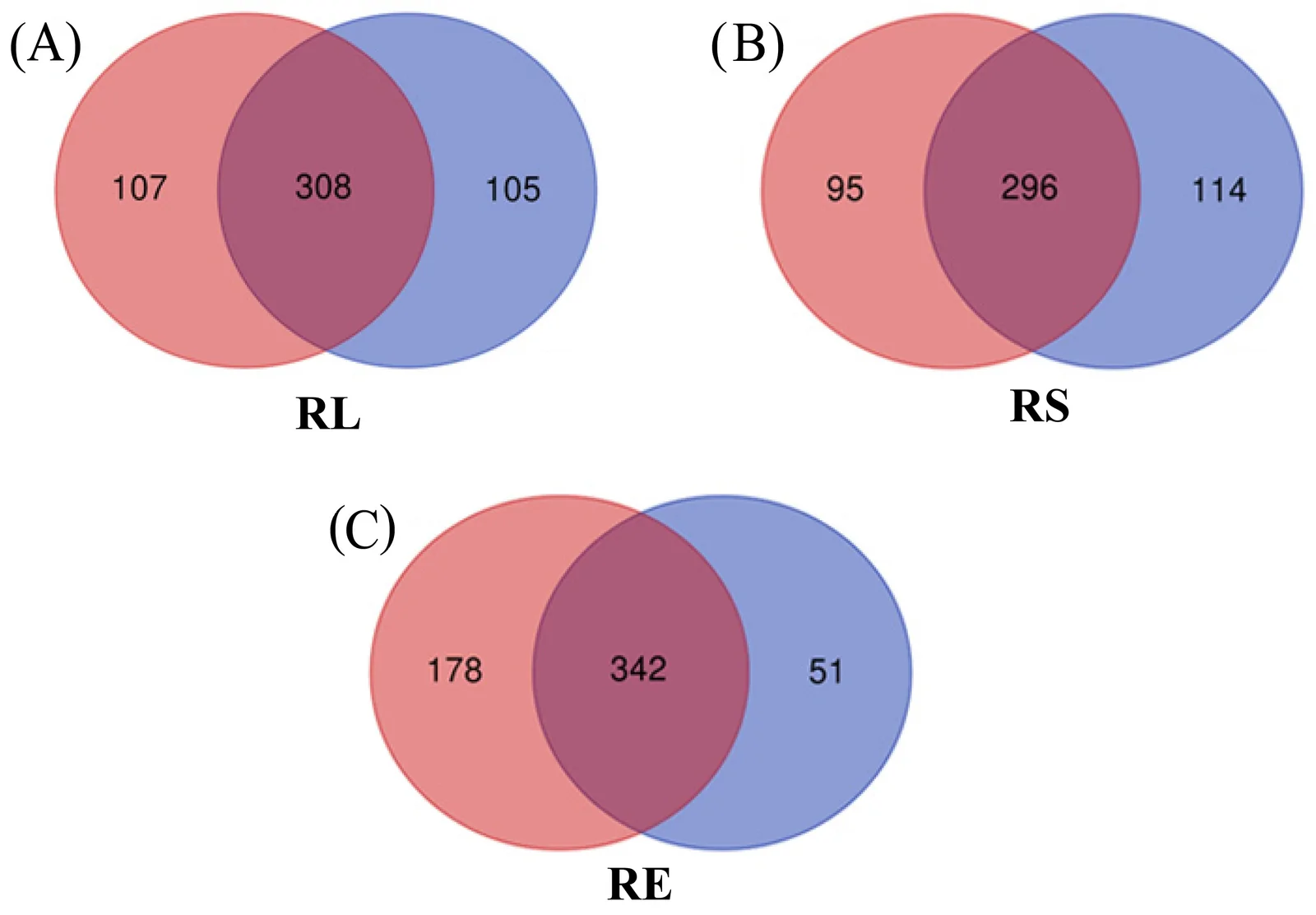
Venn diagrams presenting each sample type’s core group of OTUs (present independent of diet), as well as groups of OTUs that were unique to specific diets. Venn diagrams were constructed using only those OTUs that were present in a single sample at ≥0.1% relative abundance. (**A**) Rumen liquid samples, (**B**) rumen solid samples, and (**C**) rumen epimural samples. Red circles = high-starch diet; blue circles = low-starch diet.

We identified a core group of OTUs present in each of the sample types, independent of diet, as well as groups of OTUs that were unique to individual sample types for specific diets (Figure 5). In RL samples, a core group of 308 OTUs was found to be present regardless of diet, while high-starch samples were associated with 107 unique OTUs and low-starch samples were associated with 105 unique OTUs. In RS samples, a core group of 296 OTUs was present, independent of diet, while high- and low-starch samples were associated with 95 and 114 unique OTUs, respectively. Applying these same criteria to RE samples revealed a core group of 342 OTUs that were present for both diets types, followed by 178 OTUs that were unique to high-starch samples and just 51 OTUs that were unique to low-starch samples.

### 3.6. Firmicutes Are Most Abundant in RS, Bacteroidetes Are Most Abundant in RL, and Proteobacteria Are Only Found in RE

**Figure 6:**
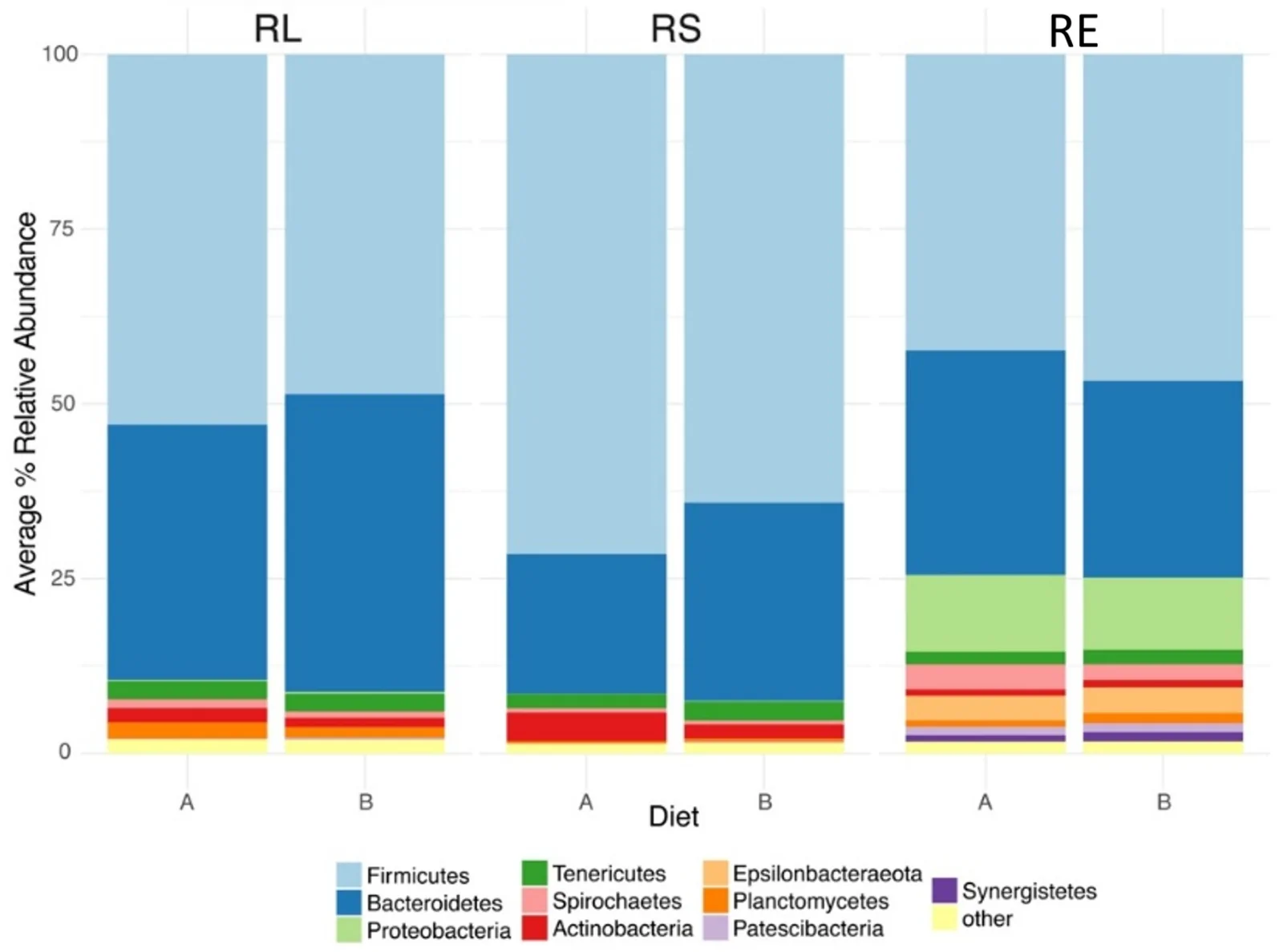
Stacked bar plot displaying the distributions of the top 10 most abundant phyla across both sample type and diet. Data presented as average % relative abundance. Sample types include rumen liquid (RL), rumen solid (RS), and epimural (RE) samples; diets include high-starch (A) and low-starch (B) diets.

Firmicutes, Bacteroidetes, and Proteobacteria were the most abundant phyla in our data set (Figure 6), and we found that, independent of diet, all sample types were dominated by Firmicutes and Bacteroidetes. Proteobacteria was also highly abundant regardless of diet, but only in RE samples. Of these phyla, for both diets, RS communities maintained the highest relative abundance of Firmicutes (RS_A = 71.5% ± 1.01; RS_B = 64.1% ± 1.42; mean ± se), and their abundance was significantly affected by diet, being higher for the high-starch treatment (*p* < 0.001). Additionally, RL communities exhibited a greater abundance of Firmicutes relative to RE samples but only for the high-starch diet (*p* < 0.001). Diet did not significantly affect the relative abundance of Firmicutes in either the RL or RE communities. Bacteroidetes were at their highest relative abundance in RL samples with the low-starch diet (RL_B = 42.6% ± 1.79); however, diet only tended (*p* = 0.08) to affect the relative abundance of Bacteroidetes in RL communities. With the high-starch diet, both the RL and RE communities had similar numbers of Bacteroidetes (RL_A = 36.5% ± 2.43; RE_A = 32.1% ± 2.81) and were significantly enriched relative to the RS community for the high-starch diet (both *p* < 0.001). However, for the low-starch diet, Bacteroidetes in RS samples rose significantly (*p* < 0.001) and became equivalent to that of RE communities, although both of these sample types were lower compared to the RL samples. Finally, Proteobacteria were only found at quantitatively significant values in RE communities (RE_A = 11.0% ± 1.07; RE_B 10.3% ± 0.66).

### 3.7. Prevotellaceae spp. Are Most Abundant in RL, Lachnospiraceae spp. Are Most Abundant in RS, and Ruminococcaceae Are Equal in RL and RS

Prevotellaceae, Lachnospiraceae, and Ruminococcaceae were the most abundant families within our data set, and all were significantly impacted by both sample type and diet treatment effects (Supplementary Table S4). Prevotellaceae were most abundant in RL sample types for the low-starch diet (RL_B = 33.1% ± 1.77). For the high-starch diet, both RL and RE samples exhibited roughly equal abundances of Prevotellaceae (RL_A = 26.3% ± 2.23; RE_A = 22.4% ± 2.74), and both of these communities maintained greater abundances than did the RS communities (both *p* < 0.01). However, for the low-starch diet, Prevotellaceae increased in relative abundance in both luminal (RS and RL) sample types (*p* < 0.05) but not in RE samples, resulting in RS and RE communities being approximately equal (RS_B = 19.6% ± 1.28; RE_B = 17.9% ± 1.23) and lower in Prevotellaceae abundance relative to the RL communities with the high-starch diet (*p* < 0.001). Independent of diet, Lachnospiraceae were highest in relative abundance in the RS sample types (RS_A = 27.7% ± 1.41; RS_B = 23.8% ± 1.15), followed by the RE samples (RE_A = 18.9% ± 0.86; RE_B = 20.5% ± 0.60), and finally the RL samples (RL_A = 14.5% ± 0.7; RL_B 15.5 ± 0.69). Diet only impacted the abundance of Lachnospiraceae in RS communities, with the high-starch diet leading to an increased abundance (*p* < 0.05). For both diet types, Ruminococcaceae were roughly equal in abundance within both luminal sample types (RL_A = 18.0% ± 0.91; RL_B = 16.1% ± 0.64; RS_A = 16.6% ± 1.07; RS_B = 17.8% ± 0.57) and were significantly less abundant in RE samples (RE_A = 6.4% ± 0.33; RE_B = 7.5% ± 0.24) (*p* < 0.001). However, diet affected the relative abundance of Ruminococcaceae in RE samples only, with the high-starch diet resulting in a higher abundance compared to the low-starch diet (*p* < 0.05).

### 3.8. Genera That Distinguish Sample Types Are Largely Conserved Across Diet, but Their Individual Contributions Differ

We used SIMPER to identify bacterial genera that contributed most to the dissimilarity between the bacterial communities associated with sample types taken from animals on the same diet (Supplementary Table S5). Within the high-starch diet, 15 different genera were implicated by our SIMPER analysis as contributing to the dissimilarity between RL and RS sample types (Supplementary Figures S1 and S2). However, with the low-starch diet, only 3 genera were identified as contributing to the dissimilarity between these two sample types. The top five genera that contributed most to the dissimilarity between groups with the high-starch diet were *Prevotella_1*, Lachnospiraceae*_NK3A20*, *Acetitomaculum*, *Rikenellaceae_RC9*, and *Mogibacterium*, while the three genera identified for the low-starch comparison corresponded to Lachnospiraceae*_NK3A20*, *Acetitomaculum*, and *Bifidobacterium* (all three of which were present in the high-starch list). With both diet types, *Prevotella_1* (*p* < 0.001) and *Rikenellaceae_RC9p* (*p* < 0.01) were more abundant in the bacterial RL communities, while Lachnospiraceae*_NK3A20* (*p* < 0.001), *Acetitomaculum* (*p* < 0.001), *Bifidobacterium* (high-starch *p* < 0.01; low-starch *p* < 0.05), and *Mogibacterium* (*p* < 0.001) were more abundant in the RS communities.

Comparing RL and RE sample types with the high-starch diet, 32 different genera were identified by our SIMPER analysis as contributing to the dissimilarity between these two sample types. With the low-starch diet this number increased to 33. Within both of these SIMPER-identified genera, the top 5 genera that contributed most to the dissimilarity between groups were the same, though the amount they contributed to the observed dissimilarities differed between diets. These genera included *Prevotella_1*, *Christensenellaceae_R.7*, *Butyrivibrio_2*, *Desulfobulbus*, and *Ruminococcaceae_NK4214*. Independent of diet, *Prevotella_1* (high-starch *p* < 0.05; low-starch *p* <0.001), *Christensenellaceae_R.7* (*p* < 0.001), and *Ruminococcaceae_NK4214* (*p* < 0.001) were more abundant in RL communities, while *Butyrivibrio_2* (*p* < 0.001) and *Desulfobulbus* (*p* < 0.001) were more abundant in RE samples.

With the high-starch diet, 31 different genera were identified by SIMPER as differing in abundance and contributing to the dissimilarity between RS and RE sample types (Supplementary Table S5). This number rose to 35 genera with the low-starch diet. Similar to the previous luminal–epimural comparison, the top 5 genera that contributed most to the dissimilarity between the RS and RE bacterial communities did not differ between diets, but their order (%–dissimilarity contribution) was different between diets. These 5 genera were *Christensenellaceae_R.7*, *Butyrivibrio_2*, *Desulfobulbus*, *Ruminococcaceae_NK4A214*, and Lachnospiraceae*_NK3A20*. Of these genera, *Christensenellaceae_R.7* (*p* < 0.001)*, Ruminococcaceae_NK4A214* (*p* < 0.001) and Lachnospiraceae*_NK3A20* (*p* < 0.001) were more abundant in RS samples, while both *Butyrivibrio_2* (*p* < 0.001) and *Desulfobulbus* (*p* < 0.001) were more abundant in RE bacterial communities.

### 3.9. Genus Level, Diet-Dependent Changes in RL, RS, and RE Bacterial Communities

We also used SIMPER to identify genus level bacterial taxa that contributed most to the effects of diet on the bacterial communities associated with different sample types (Supplementary Table S5). According to this analysis, 12 different genera were identified by SIMPER as being both differentially abundant and contributing substantially to the dissimilarity between diet treatment groups in the RL samples (Supplementary Figures S1 and S2). The top 5 of these dissimilarity-contributing and differentially abundant RL genera were *Prevotella_1*, *Ruminococcus_2*, Lachnospiraceae*_NK3A20*, Lachnospiraceae*_XPB1014*, and *p.1088.a5* (Pirellulaceae). Of these groups, *Ruminococcus_2* (*p* < 0.05), Lachnospiraceae*_NK3A20* (*p* < 0.01) *and p.1088.a5* (*p* < 0.05) were increased in relative abundance with the high-starch diet, while *Prevotella_1* (*p* < 0.01) and Lachnospiraceae*_XPB1014* (*p* < 0.05) increased with the low-starch diet.

Within the RS communities, 22 different genera were identified by SIMPER as being both differentially abundant and contributing significantly to the dissimilarity between diet treatment groups. The top 5 of these genera were *Prevotella_1*, *Christensenellaceae_R.7*, Lachnospiraceae*_NK3A20*, *Acetitomaculum*, and *F082_ge*. Of these genera, *Christensenellaceae_R.7* (*p* < 0.001), Lachnospiraceae*_NK3A20* (*p* < 0.01), *and Acetitomaculum* (*p* < 0.001) were increased in relative abundance with the high-starch diet, while *Prevotella_1* (*p* < 0.001) and *F082_ge* (*p* < 0.001) were increased with the low-starch diet.

Regarding the RE communities, 12 different genera were identified by our SIMPER analysis as being both differentially abundant and contributing significantly to the dissimilarity between diet treatment groups (Supplementary Table S5). The five genera that contributed most to the diet-dependent differences in epimural bacterial communities and that differed significantly in relative abundance in response to diet treatment were *Succiniclasticum*, *Succinivibrionaceae_UCG.001*, *Christensenellaceae_R.7*, *Desulfovibrio*, and *Family_XIII_AD3011.* Here, only *Succinivibrionaceae_UCG.001* (*p* < 0.001) increased in relative abundance with the high-starch diet, while *Succiniclasticum* (*p* < 0.05), *Christensenellaceae_R.7* (*p* < 0.05), *Desulfovibrio* (*p* < 0.01) and *Family_XIII_AD3011* (*p* < 0.01) were all increased with the low-starch diet.

## 4. Discussion

In this study, we used 16S rRNA bacterial sequencing to characterize the bacterial communities associated with the rumen (liquid, solid, and epimural) and explored how diet affects these microbiotas. Given the effects that diet is known to have on the overall environment of the rumen (VFA profiles and pH) [29] and the known differences in the spatial location of the epimural microbiota at the point of VFA absorption by the host [30,31], we hypothesized that diet would lead to significant changes in not only the RL and RS bacterial communities but also the RE communities.

We found that, while diet did not significantly impact the total abundance of VFAs, diet did impact the molar proportions of propionate, valerate, and acetate. In general, the diet-dependent differences in the overall VFA profiles we observed are congruent with the literature [29] and likely reflect changes in the bacterial communities and, subsequently, changes in the types of fermentation occurring within the rumen environment [32]. This is further reflected in the previously described differences in DMI between these cohorts [16] as a result of this dietary shift and may also be reflected in our observed changes in the ruminal microbiotas.

Within sample types, the RS bacterial community was observed to increase in diversity but not in richness in response to diet treatment. This suggests that the overall number of dominant RS bacterial species differs in response to diet, rather than the total number of species present, and that the low-starch diet produced a more diverse (even) RS community. This may be explained through the direct and indirect effects of diet treatment. In contrast to grain-based diets, diets rich in cellulosic plant material, such as the low-starch diet used here, are characterized by a high and diverse abundance of structural carbohydrates [29]. Subsequently, this type of diet could potentially select for a much larger range of dominant bacterial species that are able to utilize the more diverse and recalcitrant carbohydrate substrates. Indirectly, the rapid production of VFAs and lactic acid associated with the high fermentation rate of starch-based diets [33] often results in decreased pH, which has been shown to negatively impact the diversity of the resident luminal rumen microbiota [1,3,34,35] and may explain the decreased RS diversity observed in response to the high-starch diet.

Independent of diet, the RE bacterial community was consistently richer, more diverse, and less dominated by highly abundant taxa than were either the RL or RS communities. These differences in α-diversity between the luminal and epimural communities may reflect the different selective pressures acting on each community. The RS and RL microbiotas are hypothesized to be shaped principally, and more directly, by the composition and abundance of carbohydrates in the host’s diet [36,37,38]. In contrast, the epimural microbiota is believed to be shaped by host anatomical features [3,10]. This includes the diffusion of oxygen and urea across the rumen epithelium and likely represents a fairly constant selective pressure that could shape the epimural microbiota independent of diet. However, given the effects that VFAs are known to have on bacterial communities [39] and the diet-dependent differences in VFA profile observed in this study, it is possible that diet may still exert some indirect influences on the epimural bacterial community. Ultimately, this higher diversity of selective pressures placed on RE microbes may be partially responsible for the greater epimural diversity we observed.

Between sample types, our results are largely in agreement with similar 16S rRNA-based studies [7,14]. These studies suggest that both sample type and diet treatment result in significantly different RL, RS, and RE bacterial communities. Importantly, the only other study to consider all three ruminal microbial communities found that, independent of diet, each sample type was associated with its own distinct bacterial communities and that all communities changed significantly in response to diet treatment [14], supporting our results. However, that study further suggested that all three bacterial communities respond in a similar magnitude to changes in diet, with no one community changing more than the others. They posited that diet can impact the variability of the epimural-associated bacterial communities, with low-starch diets resulting in a less varied and more well-defined microbiota.

The differences we observed in RE community variability are likely best explained through diet indirectly influencing the epimural microbiota. Specifically, the rate, degree, and products of ruminal fermentation are known to differ by diet [29,40], and these diet-dependent parameters may have an effect on epimural microbes. For example, the slower rate of fermentation typical of high-forage (low-starch) diets [29] may result in a much more consistent release of fermentation products, generating a more stable epimural environment. This is in contrast to the rapid breakdown and release of fermentation products attributed to high-starch diets. Moreover, ruminal pH (largely a function of VFA concentration) can affect the rate of VFA uptake by rumen epithelium [31,41] and subsequently the diffusion of blood-bound oxygen, urea, and bicarbonate into the rumen, with a lower pH being associated with a higher VFA uptake and more diffusion of blood-bound compounds. As a result, it is possible that diet is indirectly influencing the epimural microbiota via the intermediate of VFA–blood metabolite exchange, with higher VFA uptake being associated with higher epimural perturbation and, subsequently, a more varied epimural microbiota. It is also possible that the chemical products generated during the fermentation of low-starch diets may simply select for a less-varied epimural community.

Regardless of diet, all sample types exhibited high abundances of Bacteroidetes and Firmicutes. Proteobacteria were also highly abundant, independent of diet, but only within RE samples. These results are in line with other research that has characterized the different bacterial communities associated with the rumen environment [7]. Within RS communities, Bacteroidetes increased in relative abundance in response to low-starch diets. However, Bacteroidetes reached their greatest abundance in the RL of forage-fed animals. The differential distribution of Bacteroidetes was closely mirrored by the distribution of Prevotellaceae and *Prevotella_1*, the most abundant family and genus within this phylum. Additionally, *Prevotella_1* was found to be an important factor in distinguishing RL samples from both RS and RE ones. This dominance in RL communities, particularly with the low-starch diet, may be a product of the metabolic flexibility of this genus.

The genus *Prevotella* is a Gram-negative, obligately anaerobic group known for its metabolic diversity and numerical dominance in the rumen environment [42,43]. Members of this group are able to grow on a variety of different sugars, amino acids, and small peptides and generally produce acetic and succinic acids from the fermentation of these sugars [42,44]. Moreover, recent research suggests that *Prevotella* spp. have a limited ability to utilize hemicelluloses [45]. As the host diet is degraded within the solid phase of the rumen, a variety of different sugar and protein by-products are released [3,30]. These diverse products diffuse into the ruminal liquid phase where they become accessible to RL bacteria. Subsequently, there may be a potential selective advantage favoring RL bacteria, such as *Prevotella* spp., which are able to use a wide range of different substrates. Further, the enrichment of *Prevotella_1* in the luminal communities of forage-fed animals potentially reflects the greater diversity of fermentative by-products, including hemicellulosic components, generated during the breakdown of this structurally complex carbohydrate diet.

Firmicutes, Lachnospiraceae, and the RS-defining genus Lachnospiraceae*_NK3A20* were always most abundant in RS samples where they were significantly enriched with the high-starch diet. Likewise, *Acetitomaculum*, another genus within the Lachnospiraceae, was also an important variable in distinguishing RS from RL and RE sample types and increased in RS samples with the high-starch diet. Lachnospiraceae_*NK3A20* has previously been shown to be a dominant bacterial member of the rumen and encodes multiple polysaccharide-degrading carbohydrate active enzymes, the majority of which are thought to target xylan [46]. The degradation of xylan is a critical function of solid-phase rumen bacteria and possibly explains the increased abundance of Lachnospiraceae*_NK3A20* within RS samples. However, it should also be noted that various members of Lachnospiraceae are known to utilize simple sugars for energy [47], which may explain why Lachnospiraceae*_NK3A20* increases in abundance with the high-starch diet. *Acetitomaculum* is an obligately anaerobic genus capable of fermenting glucose, cellobiose, fructose and esculin to acetate [48]. This genus is also able to perform acetogenesis; however, given the competition for hydrogen with ruminal methanogens, in vivo acetogenesis by *Acetitomaculum* is unlikely [49]. Subsequently, the metabolism of ruminal *Acetitomaculum* may possibly favor catabolic sugar pathways. As such, the liberation of simple sugars from plant fibers within the RS environment, and the increased abundance of such sugars within the high-starch diet, could potentially produce an environment in which *Acetitomaculum* can thrive.

Proteobacteria, the third most abundant phylum within our data set, was found almost exclusively in the RE microbiota and was unaffected by diet. This is consistent with the results of AlZahal et al. [14], who found that sample type, but not diet, impacted the abundance of Proteobacteria. Within this phylum, *Butyrivibrio_2* and *Desulfobulbus* contributed most to the dissimilarity between RE and both luminal sample types, suggesting that, regardless of diet, they are important variables in distinguishing the RE from the RL and RS. However, the observation that neither of these genera, nor Proteobacteria in general, differed in RE abundance as a consequence of diet suggests that they may not be meaningful factors in distinguishing the epimural communities of starch- and forage-fed animals. In contrast, *Succiniclasticum*, a member of the Firmicutes, was found to be an important variable in both defining the RE bacterial community and in distinguishing RE communities under different diet types.

The most abundant *Desulfobulbus* OTU in this data set, OTU4, shares 98% sequence identity with the 16S rRNA gene from *Desulfobulbus oligotrophicus* and 97% sequence identity to *D. propionicus*. Both of these species, and the genus in general, are anaerobic chemoorganotrophs capable of oxidizing propionate, lactate, and pyruvate, in the presence of a sulfur-containing electron acceptor, to a mixture of acetate and CO_2_ [50,51]. However, recent work has demonstrated that *D. propionicus* can use oxygen as an electron acceptor in the presence of sulfur-containing compounds [52]. Additionally, lactate and pyruvate can be fermented by *Desulfobulbus* to a mixture of propionate and acetate [50,51]. Given these metabolic functions, and the epimural-favored differential distribution of *Desulfobulbus*, the metabolism of fermentative by-products such as lactate, pyruvate, and other VFAs may be an additional function of the epimural microbiota. Further, the metabolic flexibility and potential aerotolerant nature of *Desulfobulbus* spp. may explain why they are unaffected by diet.

In contrast, *Succiniclasticum*, a genus originally isolated from the rumen, is characterized by being unable to ferment carbohydrates, amino acids, or carboxylic acids other than succinate, which is converted to propionate [53]. During a feed-induced rumen acidosis study, Petri et al. [54] identified epimural *Succinicalsticum* as being enriched during a subacute rumen acidosis challenge induced through a high-starch diet. This is in contrast to our results, which show that epimural *Succiniclasticum* increases in abundance with the low-starch diet. This observation could be explained via the time, relative to feeding, at which rumen samples were taken. Our rumen samples were taken 2 h prior to the morning feed, whereas Petri et al. [54] sampled animals 4 h post feeding. Given that high-starch diets are more rapidly and completely fermented than forage, the available succinate, the principal substrate for *Succinicalsticum*, could have been lower in starch-fed animals at our sampling time, relative to forage-fed animals. Additionally, the diet-dependent differences in propionate, the sole metabolic product of ruminal *Succinicalsticum*, may have influenced the efficiency of succinate fermentation by this genus, with higher propionate leading to decreased fitness and lower abundances in starch-fed animals. These differences highlight the need for further investigation into how epimural communities differ over time, particularly with respect to feeding. Regardless, the observed RE-favored abundance of *Succinicalsitcum*, along with its metabolically narrow capabilities, further suggests VFA metabolism as a potential role of the epimural microbiota and may explain why this microbe responds significantly in response to diet.

We note that the genus-level functional taxonomic group *Prevotellaceae_UCG.001* did not follow the general distribution pattern of *Prevotella_1* or its parent family Prevotellaceae and was instead more abundant in RE samples than it was in either luminal sample type. This suggests that *Prevotellaceae_UCG.001* might be selected for in the epimural environment and may represent a true epimural resident. However, given the metabolic flexibility of *Prevotella* spp. and the poorly defined character of *Prevotellaceae_UCG.001*, it is difficult to assess what role this microbe may be fulfilling within the epimural microbiota.

## 5. Conclusions

In conclusion, the major ruminal sample types (liquid, solid, and epimural) are characterized by bacterial communities exhibiting differing microbial distributions, with the epimural community being generally the most diverse and rich. Moreover, we found that each of these communities can be significantly affected by host diet. While the structure and composition of each community appears to be equally affected by diet, the evenness of rumen solids is uniquely impacted, with low-starch diets selecting for a more even community. Furthermore, at least with respect to the present experiment, low-starch diets can decrease the variability of the epimural microbiota and produce a more well-defined bacterial community. Given that many of the bacterial genera that define the epimural microbiota and respond significantly to changes in diet are likely involved in the metabolism of fermentative by-products, such as lactic acid and VFAs, the utilization of such by-products may be an underappreciated role of epimural microbes. As such, the composition and abundance of products generated during the luminal fermentation of different diets may play a role in shaping the impact of diet on the epimural community. Potential areas of future research include exploring how the epimural community changes over time, particularly with respect to time of feeding; further characterization of certain members of the epimural community, including uncultured groups of Prevotellaceae and Succinivibrionaceae; and the use of other methodological approaches (e.g., metagenomics, metatranscriptomics, metabolomics, etc.) to provide more detailed insights into the functional role of the epimural microbiome. Finally, we note some limitations inherent in our study, which include the lack of relevant physiological indicators, such as ruminal epithelial morphology, and the inability of 16S rRNA data to provide insights into the functional attributes of the ruminal microbiome.

## Data Availability

The original contributions presented in this study are included in this article and Supplementary Materials. Raw sequence data generated as part of this study are publicly available through the NCBI’s Short Read Archive under accession number PRJNA562284. Further inquiries can be directed to the corresponding author.

## Supplementary Materials

The following supporting information can be downloaded at https://www.mdpi.com/article/10.3390/microorganisms14091968/s1, Figure S1: Box plots displaying the relative abundance of the top 16 genera across each sample type and diet. RL = ruminal liquid; RS = ruminal solid; RE = ruminal epimural; Figure S2: Box plot displaying the relative abundance of the top 16 families across each sample type and diet. RL = ruminal liquid; RS = ruminal solids; RE = ruminal epimural; Table S1: Composition of both experimental diets (high-starch and low-starch) for both experimental periods (Period 1 and Period 2) and their chemical analyses (average +/− SD); Table S2: Complete PCR primer sequences used for multiplexing of samples that include Illumina sequencing adapters (MiSeq-specific), unique barcodes, and 515F/806R-specific universal bacterial 16S rRNA primers; Table S3: Volatile fatty acid concentrations (mmol/L) from samples obtained from each cow after diet shift in this cross-over design study where 7 cows were subjected to a low-starch diet followed by a high-starch diet and 6 cows were subjected to a high-starch diet; Table S4: Relative abundances (mean ± SE) of major bacterial taxa across ruminal sampling locations (RL = rumen liquid; RS = rumen solid; RE = rumen epithelium) and diets (high-starch and low-starch). Two groups of superscripts (high-starch = a, b, c and low-starch = x, y, z) are used to denote significant differences (*p* ≤ 0.05).; Table S5: Relative abundances of major bacterial taxa found to be enriched between sampling location (RL = rumen liquid; RS = rumen solid; RE = rumen epithelium) across diet as determined by SIMPER.

## Abbreviations

The following abbreviations are used in this manuscript:

DMI	Dry Matter Intake
DIM	Days in Milk
OTU	Operational Taxonomic Unit
PERMANOVA	Permutational ANOVA
RL	Ruminal Liquid
RS	Ruminal Solid
RE	Ruminal Epithelium
SIMPER	Similarity Percentage
VFA	Volatile Fatty Acid
