# Supplementary material for: Evaluating the Impact of High- and Low-Starch Diets on the Ruminal Microbiota of Dairy Cows
Source: Microorganisms. 2026 Sep 6;14(9):1968. doi: 10.3390/microorganisms14091968 (PMC13609410; doi:10.3390/microorganisms14091968)
Supplement: Supplementary file 1 [file microorganisms-14-01968-s001.zip › microorganisms-4513739-supplementary.pdf]

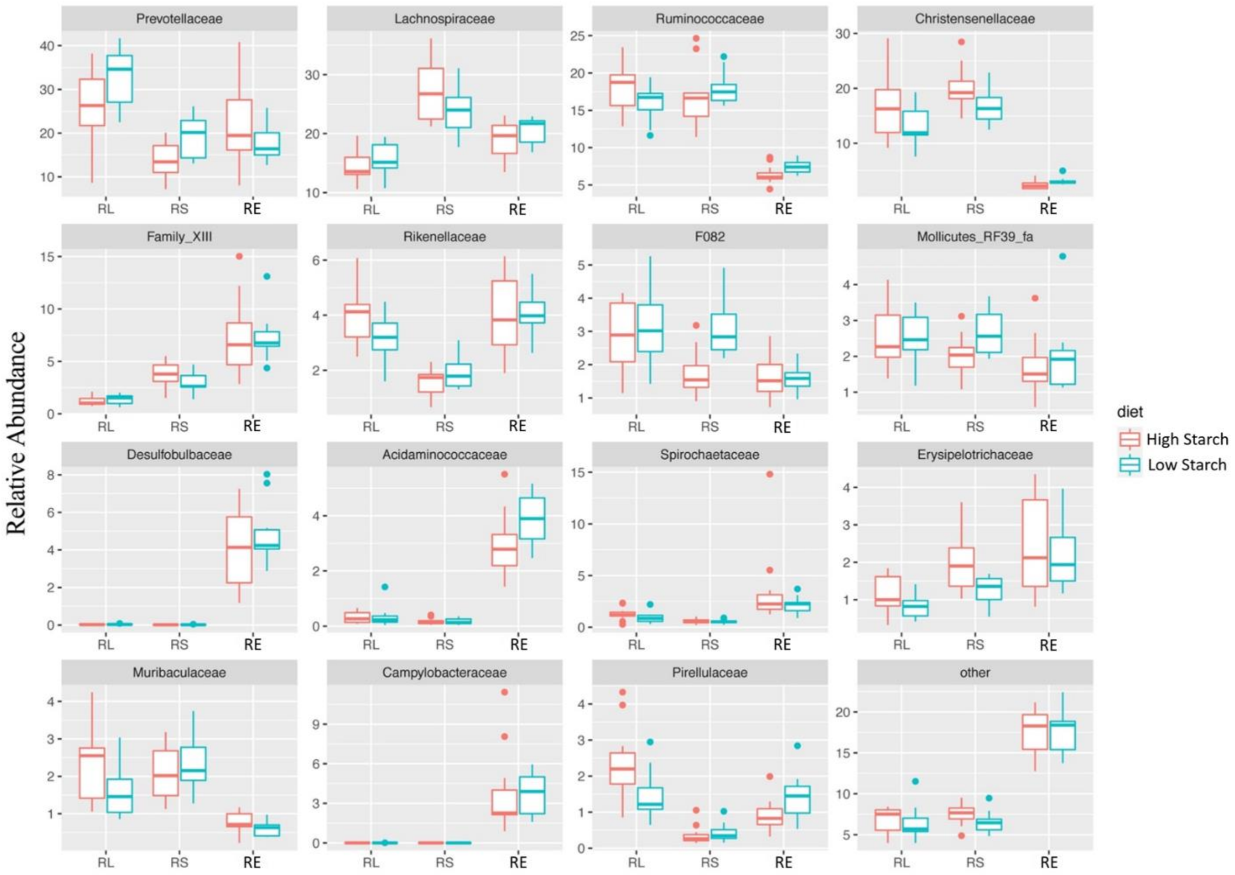

Supplemental Figure S1: Box plots displaying the relative abundance of the top 16 genera across each sample-type and diet. RL = ruminal liquid; RS = ruminal solid; RE = ruminal epimural

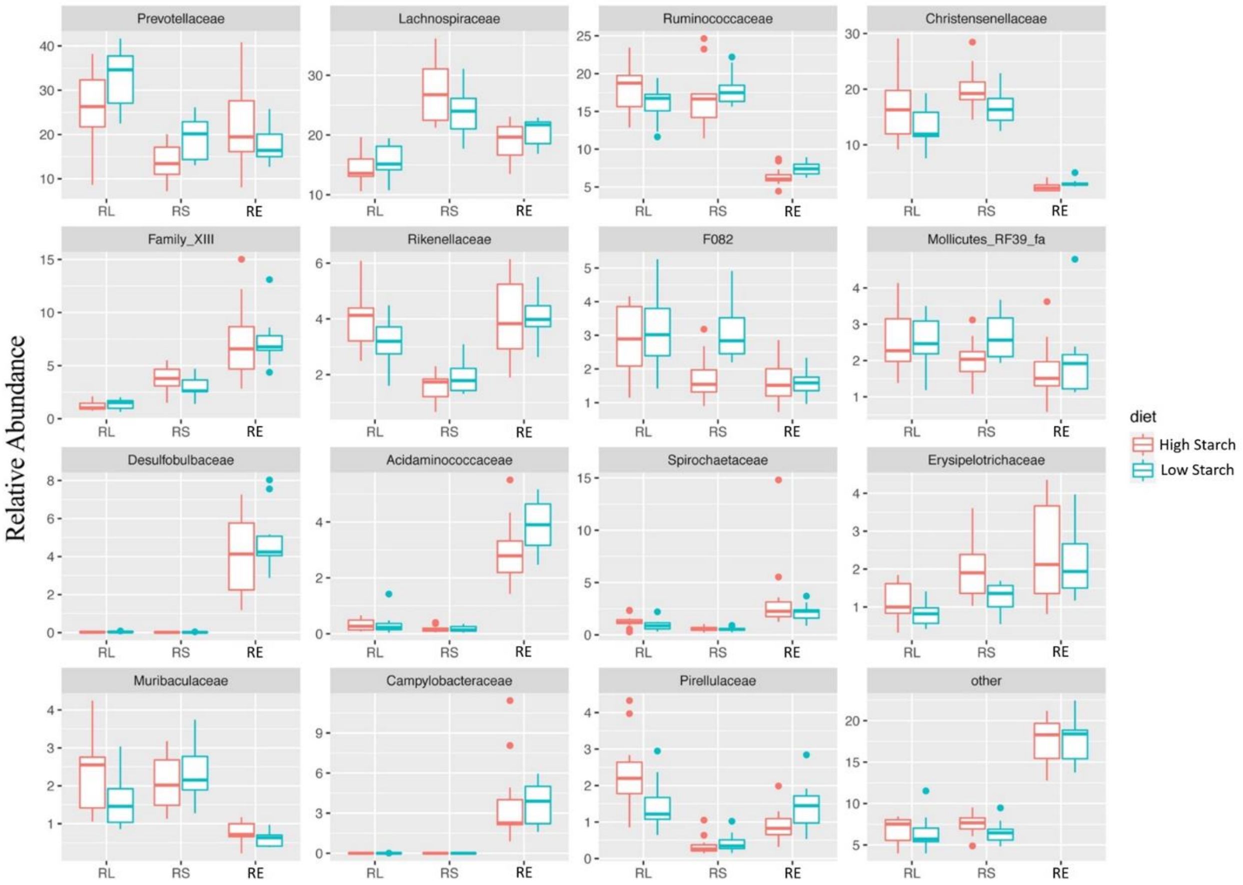

Supplemental Figure S2: Box plot displaying the relative abundance of the top 16 families across each sample-type and diet. RL = ruminal liquid; RS = ruminal solids; RE = ruminal epimural

Supplemental Table S1: Composition of both experimental diets (high-starch and low-starch) for both experimental periods (Period 1 and Period 2) and their chemical analyses (average +/- SD.

	High Starch		Low Starch	
	Period 1	Period 2	Period 1	Period 2
Ingredients (% of diet DM[1])				
Alfalfa Silage	24.2 +/- 0.66	24.1 +/- 0.54	33.9 +/- 0.79	33.9 +/- 0.66
Corn Silage	23.2 +/- 0.42	23.0 +/- 0.41	32.5 +/- 0.59	32.4 +/- 0.55
High moisture corn	24.5 +/- 0.41	25.0 +/- 0.31	0	0
Beet Pulp pelleted	2.8 +/- 0.04	2.7 +/- 0.02	8.9 +/- 0.14	8.9 +/- 0.11
Canola Meal	9.7 +/- 0.13	9.8 +/- 0.12	2.6 +/- 0.05	2.7 +/- 0.04
Corn Distillers Grain	2.7 +/- 0.04	2.6 +/- 0.03	9.2 +/- 0.23	9.2 +/- 0.12
Roasted soybean	4.1 +/- 0.05	4 +/- 0.03	4.1 +/- 0.06	4.1 +/- 0.05
Soybean Hulls	6.1 +/- 0.08	6.1 +/- 0.06	6.1 +/- 0.10	6.1 +/- 0.09
Mineral and Vitamin Mix[2]	2.7 +/- 0.03	2.7 +/- 0.03	2.7 +/- 0.04	2.7 +/- 0.04
Nutrient Composition (% of diet DM[1])				
DM (% of DM)	50.1 +/- 0.51	50.9 +/- 0.29	45.1 +/- 0.59	45.9 +/- 0.39
CP (% of DM)	16.2 +/- 0.08	16.0 +/- 0.08	16.5 +/- 0.09	16.5 +/- 0.10
NDF (% of DM)	29.0 +/- 0.29	28.9 +/- 0.11	36.9 +/- 0.30	36.9 +/- 0.12
Forage NDF (% of NDF[3])	61.1 +/- 0.50	61.3 +/- 0.52	67.2 +/- 0.45	67.6 +/- 0.40
ADF (% of DM)	21.1 +/- 0.14	21.0 +/- 0.13	27.5 +/- 0.11	27.4 +/- 0.16
Lignin (% of DM)	3.0 +/- 0.03	3.3 +/- 0.12	3.6 +/- 0.08	3.9 +/- 0.20
EE (% of DM)	4.9 +/- 0.22	4.8 +/- 0.01	5.1 +/- 0.23	5.1 +/- 0.06
Ash (% of DM)	5.7 +/- 0.10	5.7 +/- 0.08	7.4 +/- 0.16	7.4 +/- 0.16
Starch (% of DM)	26.7 +/- 0.29	27.5 +/- 0.31	12.8 +/- 0.18	13.0 +/- 0.27
DEp_3X (Mcal/kg DM)[4]	3.02 +/- 0.01	3.04 +/- 0.005	2.91 +/- 0.01	2.88 +/- 0.01
MEp_3X (Mcal/kg DM)[4]	2.62 +/- 0.01	2.64 +/- 0.006	2.51 +/- 0.01	2.48 +/- 0.01
NELp_3X (Mcal/kg DM)[4]	1.67 +/- 0.01	1.68 +/- 0.004	1.59 +/- 0.008	1.57 +/- 0.007

[1] SDs were calculated using the day-to-day change in offered diet composition.

[2] The mineral and vitamin mix contained (on a DM basis): 16.0% Ca, 5.85% Mg, 0.54% K, 14.8% Na, 6.67% Cl, 0.73% S, 42.5 mg of Co/kg, 519 mg of Cu/kg, 60.2 mg of I/kg, 778 mg of Fe/kg, 2 601 mg of Mn/kg,14.6 mg of Se/kg, 2 808 mg of Zn/kg, 292 kIU of vitamin A/kg, 58.5 kIU of vitamin D/kg, 1.36 kIU of vitamin E/kg, and 0.494 g of monensin/kg (Vita Plus Corporation, Madison, WI).

[3] Forage NDF is reported as a percentage of NDF.

[4] Digestible energy (DEp_3X), metabolizable energy (MEp_3X) and net energy (NELp_3X) at production level were calculated according to NRC (2001) equations, considering that the cows are eating 3 times the maintenance requirements. Those variables were reported in Mcal/kg DM.

Supplemental Table S2: Complete PCR primer sequences used for multiplexing of samples that include Illumina sequencing adapters (MiSeq-specific), unique barcodes, and 515F/806R-specific universal bacterial 16S rRNA primers.

Sequence Name	Direction	Sequence (5' to 3')
SA501	Forward	AAT GAT ACG GCG ACC ACC GAG ATC TAC ACA TCG TAC GTA TGG TAA TTG TGT GCC AGC MGC CGC GGT AA
SA502	Forward	AAT GAT ACG GCG ACC ACC GAG ATC TAC ACA CTA TCT GTA TGG TAA TTG TGT GCC AGC MGC CGC GGT AA
SA503	Forward	AAT GAT ACG GCG ACC ACC GAG ATC TAC ACT AGC GAG TTA TGG TAA TTG TGT GCC AGC MGC CGC GGT AA
SA504	Forward	AAT GAT ACG GCG ACC ACC GAG ATC TAC ACC TGC GTG TTA TGG TAA TTG TGT GCC AGC MGC CGC GGT AA
SA505	Forward	AAT GAT ACG GCG ACC ACC GAG ATC TAC ACT CAT CGA GTA TGG TAA TTG TGT GCC AGC MGC CGC GGT AA
SA506	Forward	AAT GAT ACG GCG ACC ACC GAG ATC TAC ACC GTG AGT GTA TGG TAA TTG TGT GCC AGC MGC CGC GGT AA
SA507	Forward	AAT GAT ACG GCG ACC ACC GAG ATC TAC ACG GAT ATC TTA TGG TAA TTG TGT GCC AGC MGC CGC GGT AA
SA508	Forward	AAT GAT ACG GCG ACC ACC GAG ATC TAC ACG ACA CCG TTA TGG TAA TTG TGT GCC AGC MGC CGC GGT AA
SB501	Forward	AAT GAT ACG GCG ACC ACC GAG ATC TAC ACC TAC TAT ATA TGG TAA TTG TGT GCC AGC MGC CGC GGT AA
SB502	Forward	AAT GAT ACG GCG ACC ACC GAG ATC TAC ACC GTT ACT ATA TGG TAA TTG TGT GCC AGC MGC CGC GGT AA
SB503	Forward	AAT GAT ACG GCG ACC ACC GAG ATC TAC ACA GAG TCA CTA TGG TAA TTG TGT GCC AGC MGC CGC GGT AA
SB504	Forward	AAT GAT ACG GCG ACC ACC GAG ATC TAC ACT ACG AGA CTA TGG TAA TTG TGT GCC AGC MGC CGC GGT AA
SB505	Forward	AAT GAT ACG GCG ACC ACC GAG ATC TAC ACA CGT CTC GTA TGG TAA TTG TGT GCC AGC MGC CGC GGT AA
SB506	Forward	AAT GAT ACG GCG ACC ACC GAG ATC TAC ACT CGA CGA GTA TGG TAA TTG TGT GCC AGC MGC CGC GGT AA
SB507	Forward	AAT GAT ACG GCG ACC ACC GAG ATC TAC ACG ATC GTG TTA TGG TAA TTG TGT GCC AGC MGC CGC GGT AA
SB508	Forward	AAT GAT ACG GCG ACC ACC GAG ATC TAC ACG TCA GAT ATA TGG TAA TTG TGT GCC AGC MGC CGC GGT AA
SA701	Reverse	CAA GCA GAA GAC GGC ATA CGA GAT AAC TCT CGA GTC AGT CAG CCG GAC TAC HVG GGT WTC TAA T
SA702	Reverse	CAA GCA GAA GAC GGC ATA CGA GAT ACT ATG TCA GTC AGT CAG CCG GAC TAC HVG GGT WTC TAA T
SA703	Reverse	CAA GCA GAA GAC GGC ATA CGA GAT AGT AGC GTA GTC AGT CAG CCG GAC TAC HVG GGT WTC TAA T
SA704	Reverse	CAA GCA GAA GAC GGC ATA CGA GAT CAG TGA GTA GTC AGT CAG CCG GAC TAC HVG GGT WTC TAA T
SA705	Reverse	CAA GCA GAA GAC GGC ATA CGA GAT CGT ACT CAA GTC AGT CAG CCG GAC TAC HVG GGT WTC TAA T
SA706	Reverse	CAA GCA GAA GAC GGC ATA CGA GAT CTA CGC AGA GTC AGT CAG CCG GAC TAC HVG GGT WTC TAA T
SA707	Reverse	CAA GCA GAA GAC GGC ATA CGA GAT GGA GAC TAA GTC AGT CAG CCG GAC TAC HVG GGT WTC TAA T
SA708	Reverse	CAA GCA GAA GAC GGC ATA CGA GAT GTC GCT CGA GTC AGT CAG CCG GAC TAC HVG GGT WTC TAA T
SA709	Reverse	CAA GCA GAA GAC GGC ATA CGA GAT GTC GTA GTA GTC AGT CAG CCG GAC TAC HVG GGT WTC TAA T
SA710	Reverse	CAA GCA GAA GAC GGC ATA CGA GAT TAG CAG ACA GTC AGT CAG CCG GAC TAC HVG GGT WTC TAA T
SA711	Reverse	CAA GCA GAA GAC GGC ATA CGA GAT TCA TAG ACA GTC AGT CAG CCG GAC TAC HVG GGT WTC TAA T

SA712	Reverse	CAA GCA GAA GAC GGC ATA CGA GAT TCG CTA TAA GTC AGT CAG CCG GAC TAC HVG GGT WTC TAA T
SB701	Reverse	CAA GCA GAA GAC GGC ATA CGA GAT AAG TCG AGA GTC AGT CAG CCG GAC TAC HVG GGT WTC TAA T
SB702	Reverse	CAA GCA GAA GAC GGC ATA CGA GAT ATA CTT CGA GTC AGT CAG CCG GAC TAC HVG GGT WTC TAA T
SB703	Reverse	CAA GCA GAA GAC GGC ATA CGA GAT AGC TGC TAA GTC AGT CAG CCG GAC TAC HVG GGT WTC TAA T
SB704	Reverse	CAA GCA GAA GAC GGC ATA CGA GAT CAT AGA GAA GTC AGT CAG CCG GAC TAC HVG GGT WTC TAA T
SB705	Reverse	CAA GCA GAA GAC GGC ATA CGA GAT CGT AGA TCA GTC AGT CAG CCG GAC TAC HVG GGT WTC TAA T
SB706	Reverse	CAA GCA GAA GAC GGC ATA CGA GAT CTC GTT ACA GTC AGT CAG CCG GAC TAC HVG GGT WTC TAA T
SB707	Reverse	CAA GCA GAA GAC GGC ATA CGA GAT GCG CAC GTA GTC AGT CAG CCG GAC TAC HVG GGT WTC TAA T
SB708	Reverse	CAA GCA GAA GAC GGC ATA CGA GAT GGT ACT ATA GTC AGT CAG CCG GAC TAC HVG GGT WTC TAA T
SB709	Reverse	CAA GCA GAA GAC GGC ATA CGA GAT GTA TAC GCA GTC AGT CAG CCG GAC TAC HVG GGT WTC TAA T
SB710	Reverse	CAA GCA GAA GAC GGC ATA CGA GAT TAC GAG CAA GTC AGT CAG CCG GAC TAC HVG GGT WTC TAA T
SB711	Reverse	CAA GCA GAA GAC GGC ATA CGA GAT TCA GCG TTA GTC AGT CAG CCG GAC TAC HVG GGT WTC TAA T
SB712	Reverse	CAA GCA GAA GAC GGC ATA CGA GAT TCG CTA CGA GTC AGT CAG CCG GAC TAC HVG GGT WTC TAA T

Supplemental Table S3. Volatile fatty acid concentrations (mmol/L) from samples obtained from each cow after diet shift in this cross-over design study where 7 cows were subjected to a low-starch diet followed by a high-starch diet and 6 cows were subjected to a high-starch diet.

Cow ID	Diet Sequence	Diet prior to Sampling	Acetate (mmol/L)	Propionate (mmol/L)	Isobutyrate (mmol/L)	Butyrate (mmol/L)	Isovalerate+2MB (mmol/L)	Valerate (mmol/L)	Total VFA (mmol/L)
4403	Low-starch->High-starch	Low-Starch	66.8	17.4	0.7	9.3	1.9	0.9	97.0
4403	Low-starch->High-starch	High-starch	57.3	16.1	0.8	8.6	2.2	1.0	86.0
4668	Low-starch->High-starch	Low-Starch	55.9	14.1	0.7	7.6	1.5	0.5	80.3
4668	Low-starch->High-starch	High-starch	78.9	19.9	1.0	18.7	2.8	1.1	122.5
5034	Low-starch->High-starch	Low-Starch	57.9	14.9	0.6	7.9	1.3	0.5	83.1
5034	Low-starch->High-starch	High-starch	72	27.3	0.6	10.6	2.3	1.4	114.2
5298	Low-starch->High-starch	Low-Starch	72.9	24.1	0.8	10.2	2.1	1.3	111.3
5298	Low-starch->High-starch	High-starch	58.9	26.1	0.6	8.7	2	1.4	97.7
6098	Low-starch->High-starch	Low-Starch	74.5	21.2	0.7	9.9	2.2	1.1	109.6
6098	Low-starch->High-starch	High-starch	65.4	21.5	0.8	9.1	2.5	1.0	100.4
6201	Low-starch->High-starch	Low-Starch	52.3	12.8	0.7	6.1	1.1	0.6	73.4
6201	Low-starch->High-starch	High-starch	52.6	15	0.7	7.6	1.4	0.8	78.1
6219	Low-starch->High-starch	Low-starch	61.1	14.8	0.7	7.2	1.5	0.7	85.9
6219	Low-starch->High-starch	High-starch	69.6	16.5	0.8	10.7	2.5	0.9	101.1
5020	High-starch->Low-starch	High-starch	56.4	12.8	0.9	9.7	1.6	0.7	82.1
5020	High-starch->Low-starch	Low-starch	73.2	17.7	0.9	10.6	2	0.9	105.1
5046	High-starch->Low-starch	High-starch	70.5	24.4	0.8	9.2	1.6	1.3	107.8
5046	High-starch->Low-starch	Low-starch	72.5	19.4	1.1	9.5	2.1	1.1	105.7
5053	High-starch->Low-starch	High-starch	70.6	24.3	0.9	10.4	2.2	1.1	109.6
5053	High-starch->Low-starch	Low-starch	67.9	18.5	0.7	9.5	1.9	0.9	99.5
5409	High-starch->Low-starch	Low-starch	53.2	14.1	0.8	7.9	2.3	0.6	78.9
5409	High-starch->Low-starch	High-starch	68.5	16.6	1.0	11.0	2.1	0.9	100.1
5677	High-starch->Low-starch	High-starch	60	17.8	0.7	8.5	2.0	0.7	89.8
5677	High-starch->Low-starch	Low-starch	53.8	14.1	0.7	6.5	1.9	0.6	77.6
5696	High-starch->Low-starch	High-starch	54.6	19	0.8	8.1	1.9	1.0	85.3
5696	High-starch->Low-starch	Low-starch	63.5	15.3	0.8	10.4	2.2	0.9	93.0

Supplemental Table S4: Relative abundances (mean ± SE) of major bacterial taxa across ruminal sampling locations (RL = rumen liquid; RS = rumen solid; RE = rumen epithelium) and diets (high-starch and low-starch). Two groups of superscripts (high-starch = a,b,c and low-starch = x,y,z) are used to denote significant differences ($P \leq 0.05$).

	Sample location						Significance of diet-treatment		
	RL		RS		RE				
Taxonomy	High-starch	Low-starch	High-starch	Low-starch	High-starch	Low-starch	RL	RS	RE
**Firmicutes**	52.97 ± 2.41^{a}	48.62 ± 1.91^{x}	71.48 ± 1.01^{b}	64.15 ± 1.42^{y}	42.38 ± 2.11^{c}	46.72 ± 0.93^{x}		***	
Lachnospiraceae	14.52 ± 0.7^{a}	15.54 ± 0.69^{x}	27.7 ± 1.41^{b}	23.79 ± 1.15^{y}	18.92 ± 0.86^{c}	20.53 ± 0.6^{z}		*	
Lachnospiraceae_NK3A20	3.91 ± 0.29^{a}	2.75 ± 0.16^{x}	11.3 ± 0.74^{b}	8.37 ± 0.8^{y}	1.83 ± 0.17^{c}	2.29 ± 0.2^{z}	**	**	
Lachnospiraceae_XPB1014	1.5 ± 0.22^{a}	2.6 ± 0.23^{x}	0.76 ± 0.08^{b}	2.04 ± 0.17^{y}	0.1 ± 0.02^{c}	0.22 ± 0.04^{z}	*	***	***
Howardella	0.12 ± 0.01^{a}	0.06 ± 0.01^{x}	0.03 ± 0^{b}	0.02 ± 0^{y}	2.27 ± 0.33^{c}	2.54 ± 0.24^{z}	***		
Butyrivibrio_2	0.25 ± 0.04^{a}	0.69 ± 0.09^{x}	0.3 ± 0.05^{a}	0.64 ± 0.09^{x}	4.79 ± 0.57^{b}	5.79 ± 0.48^{y}	***	**	
Ruminococcaceae	18.01 ± 0.91^{a}	16.08 ± 0.64^{x}	16.59 ± 1.07^{a}	17.85 ± 0.57^{x}	6.39 ± 0.33^{b}	7.47 ± 0.24^{y}			*
Ruminococcus_2	3.46 ± 0.83^{a}	1.62 ± 0.25^{a}	3.64 ± 0.89^{a}	2.6 ± 0.3^{b}	0.69 ± 0.16^{b}	0.37 ± 0.05^{c}	*		
Ruminococcaceae_NK4A214	6.71 ± 0.68^{a}	6.18 ± 0.39^{x}	6.03 ± 0.37^{a}	5.68 ± 0.25^{x}	1.29 ± 0.11^{b}	1.59 ± 0.08^{y}			
Saccharofermentans	1.86 ± 0.1^{a}	1.76 ± 0.08^{x}	2.06 ± 0.14^{a}	2.42 ± 0.1^{y}	0.33 ± 0.06^{b}	0.39 ± 0.05^{z}		*	
Christensenellaceae	16.27 ± 1.58^{a}	13.32 ± 1.08^{x}	19.98 ± 1.1^{b}	16.88 ± 0.95^{y}	2.37 ± 0.2^{c}	3.07 ± 0.18^{z}		***	*
Christensenellaceae_R-7	16.14 ± 1.56^{a}	13.12 ± 1.07^{x}	19.72 ± 1.08^{b}	16.5 ± 0.93^{y}	2.16 ± 0.2^{c}	2.73 ± 0.17^{z}		***	*
Family_XIII	1.2 ± 0.12^{a}	1.36 ± 0.13^{x}	3.81 ± 0.3^{b}	2.9 ± 0.26^{y}	7.24 ± 0.99^{c}	7.23 ± 0.59^{z}		*	
Acidaminococcaceae	0.32 ± 0.06^{a}	0.32 ± 0.1^{x}	0.16 ± 0.03^{b}	0.17 ± 0.03^{y}	2.92 ± 0.32^{c}	3.86 ± 0.25^{z}			*
Succiniclasticum	0.32 ± 0.06^{a}	0.32 ± 0.1^{x}	0.16 ± 0.03b	0.17 ± 0.03^{y}	2.9 ± 0.32^{c}	3.86 ± 0.25^{z}			*
**Bacteriodetes**	36.51 ± 2.43^{a}	42.57 ± 1.79^{x}	19.98 ± 1.15^{b}	28.32 ± 1.4^{y}	32.09 ± 2.82^{a}	28.15 ± 1.14^{y}		***	
Prevotellaceae	26.34 ± 2.23^{a}	33.13 ± 1.77^{x}	13.8 ± 1.07^{b}	19.63 ± 1.28^{y}	22.44 ± 2.74^{a}	17.92 ± 1.23^{y}	*	***	
Prevotella_1	20.21 ± 1.86^{a}	27.95 ± 1.73^{x}	10.22 ± 0.87^{b}	15.34 ± 1.13^{y}	13.66 ± 2.11^{b}	10.8 ± 1.06^{z}	**	***	
Prevotellaceae_UCG-001	1.27 ± 0.15^{a}	1.4 ± 0.14^{x}	1.25 ± 0.11^{a}	1.78 ± 0.13^{y}	4.07 ± 0.25^{b}	3.62 ± 0.24^{z}		**	

Rikenellaceae	3.93 ± 0.27^{a}	3.15 ± 0.24x	1.59 ± 0.13^{b}	1.9 ± 0.15y	3.88 ± 0.37^{a}	4.13 ± 0.22^{z}	*		
Rikenellaceae_RC9	3.71 ± 0.26^{a}	2.95 ± 0.21x	1.53 ± 0.13^{b}	1.8 ± 0.15^{y}	3 ± 0.29^{c}	3.42 ± 0.18^{x}	*		
**Proteobacteria**	0.17 ± 0.02^{a}	0.26 ± 0.02^{x}	0.06 ± 0.01^{b}	0.15 ± 0.03^{y}	10.98 ± 1.07^{c}	10.32 ± 0.66^{z}			
Desulfobulbaceae	0.03 ± 0^{a}	0.04 ± 0.01^{x}	0.01 ± 0^{b}	0.02 ± 0^{y}	4.12 ± 0.55^{c}	4.75 ± 0.41^{z}	*	***	
Desulfobulbus	0.03 ± 0^{a}	0.04 ± 0.01^{x}	0.01 ± 0^{b}	0.02 ± 0^{y}	4.12 ± 0.55^{c}	4.75 ± 0.41^{z}			
Succinivibrionaceae	0.02 ± 0^{a}	0.05 ± 0.01^{x}	0.01 ± 0^{a}	0.04 ± 0.02^{y}	1.88 ± 0.48^{b}	0.9 ± 0.09^{z}	*		
Succinivibrionaceae_UCG.001	0.01 ± 0^{a}	0 ± 0^{x}	0 ± 0^{a}	0 ± 0^{x}	1.26 ± 0.43^{b}	0.09 ± 0.04^{y}			***
Succinivibrionaceae_UCG.002	0.01 ± 0^{a}	0.04 ± 0.01^{x}	0 ± 0^{a}	0.03 ± 0.02^{x}	0.41 ± 0.18^{b}	0.62 ± 0.08^{y}	**		***
Desulfovibrionaceae	0 ± 0^{a}	0.01 ± 0^{x}	0 ± 0^{a}	0 ± 0^{y}	0.58 ± 0.14^{b}	0.91 ± 0.12^{z}			
Desulfovibrio	0 ± 0^{a}	0.01 ± 0^{x}	0 ± 0^{a}	0 ± 0^{y}	0.58 ± 0.14^{b}	0.9 ± 0.12^{z}	**		**
Burkholderiaceae	0.01 ± 0^{a}	0.01 ± 0^{x}	0 ± 0^{a}	0 ± 0^{x}	1.23 ± 0.25^{b}	0.85 ± 0.14^{y}			
Comamonas	0.01 ± 0^{a}	0 ± 0^{x}	0 ± 0^{b}	0 ± 0^{x}	1.21 ± 0.25^{c}	0.81 ± 0.14^{y}			
Cardiobacteriaceae	0.04 ± 0.01^{a}	0.02 ± 0^{x}	0 ± 0^{b}	0 ± 0^{y}	1.38 ± 0.47^{c}	1.35 ± 0.3^{z}			
**Spirochaetes**	1.27 ± 0.16^{a}	0.91 ± 0.14^{x}	0.6 ± 0.07^{b}	0.54 ± 0.05^{y}	3.59 ± 1.01^{c}	2.27 ± 0.23^{z}			
Treponema_2	1.26 ± 0.16^{a}	0.89 ± 0.14^{x}	0.6 ± 0.07^{b}	0.54 ± 0.05^{y}	3.36 ± 1^{c}	2.02 ± 0.22^{z}			
**Epsilonbacteraeota**	0 ± 0^{a}	0 ± 0^{x}	0 ± 0^{a}	0 ± 0^{x}	3.53 ± 0.84^{b}	3.7 ± 0.44^{y}			
Campylobacter	0 ± 0^{a}	0 ± 0^{x}	0 ± 0^{a}	0 ± 0^{x}	3.53 ± 0.84^{b}	3.7 ± 0.44^{y}			
**Tenericutes**	2.59 ± 0.23^{a}	2.54 ± 0.19^{x}	2.02 ± 0.14^{b}	2.7 ± 0.18^{x}	1.81 ± 0.21^{b}	2.05 ± 0.27^{x}		*	
**Planctomycetes**	2.36 ± 0.26^{a}	1.51 ± 0.17^{x}	0.35 ± 0.07^{b}	0.43 ± 0.07^{y}	0.9 ± 0.12^{c}	1.43 ± 0.17^{x}	*		***
p-1088-a5	1.56 ± 0.13^{a}	1.07 ± 0.13^{x}	0.26 ± 0.06^{b}	0.36 ± 0.06^{y}	0.5 ± 0.06^{c}	0.84 ± 0.1^{z}	*		**

Supplemental Table S5: Relative abundances of major bacterial taxa found to be enriched between sampling location (RL = rumen liquid; RS = rumen solid; RE = rumen epithelium) across diet as determined by SIMPER.

Sample Location Comparison	Diet	Taxon	Relative Sequence Abundance (%)	SD	*P*-value
RL x RS	High Starch	Prevotella_1	0.053	0.030	0.003
		Lachnospiraceae_NK3A20_group	0.037	0.014	0.001
		Acetitomaculum	0.012	0.010	0.001
		Rikenellaceae_RC9_gut_group	0.011	0.005	0.001
		Mogibacterium	0.008	0.003	0.002
		F082_ge	0.007	0.004	0.002
		p.1088.a5_gut_group	0.006	0.002	0.001
		Bifidobacterium	0.005	0.004	0.013
		CPla.4_termite_group	0.004	0.003	0.002
		Moryella	0.003	0.002	0.009
		Family_XIII_AD3011_group	0.003	0.002	0.001
		Syntrophococcus	0.003	0.002	0.001
		Lachnospiraceae_FE2018_group	0.002	0.001	0.001
		WCHB1.41_ge	0.002	0.002	0.012
		Marvinbryantia	0.002	0.001	0.001
	Low Starch	Lachnospiraceae_NK3A20_group	0.019	0.016	0.018
		Acetitomaculum	0.002	0.002	0.005
		Bifidobacterium	0.001	0.001	0.006
RL x RE	High Starch	Christensenellaceae_R.7_group	0.070	0.027	0.001
		Prevotella_1	0.049	0.033	0.017
		Ruminococcaceae_NK4A214_group	0.027	0.012	0.001
		Butyrivibrio_2	0.023	0.010	0.001
		Desulfobulbus	0.020	0.009	0.001
		Campylobacter	0.018	0.015	0.001
		Prevotellaceae_UCG.001	0.014	0.005	0.001
		Succiniclasticum	0.013	0.006	0.001
		unclassified	0.012	0.009	0.003
		Family_XIII_ge	0.012	0.009	0.001
		uncultured	0.012	0.006	0.001
		Howardella	0.011	0.006	0.001
		Ruminococcaceae_UCG.014	0.010	0.006	0.001
		Mogibacterium	0.009	0.006	0.001
		Saccharofermentans	0.008	0.002	0.001
		Lachnospiraceae_UCG.008	0.007	0.004	0.001

		Muribaculaceae_ge	0.007	0.005	0.001
		F082_ge	0.007	0.005	0.001
		Lachnospiraceae_XPB1014_group	0.007	0.004	0.001
		Comamonas	0.006	0.004	0.001
		Mollicutes_RF39_ge	0.006	0.004	0.003
		p.1088.a5_gut_group	0.005	0.002	0.001
		JGI_0000069.P22_ge	0.005	0.004	0.001
		uncultured_ge	0.005	0.001	0.001
		Flexilinea	0.004	0.002	0.001
		Ruminococcaceae_ge	0.004	0.003	0.001
		Anaerovorax	0.004	0.003	0.001
		Fibrobacter	0.004	0.002	0.001
		Fretibacterium	0.004	0.002	0.001
		Family_XIII_AD3011_group	0.003	0.002	0.002
		WCHB1.41_ge	0.003	0.002	0.001
		U29.B03	0.002	0.002	0.006
		Oribacterium	0.002	0.002	0.008
		Lachnospiraceae_UCG.010	0.002	0.001	0.001
		Lachnospira	0.002	0.007	0.019
	Low Starch	Prevotella_1	0.063	0.041	0.001
		Christensenellaceae_R.7_group	0.057	0.020	0.001
		Butyrivibrio_2	0.024	0.009	0.001
		Desulfobulbus	0.023	0.008	0.001
		Ruminococcaceae_NK4A214_group	0.023	0.006	0.001
		Campylobacter	0.018	0.008	0.001
		Succiniclasticum	0.018	0.004	0.001
		unclassified	0.012	0.008	0.006
		uncultured	0.011	0.004	0.001
		Prevotellaceae_UCG.001	0.011	0.005	0.001
		Lachnospiraceae_XPB1014_group	0.011	0.004	0.001
		Howardella	0.010	0.004	0.001
		Ruminococcaceae_UCG.014	0.010	0.005	0.001
		Saccharofermentans	0.009	0.002	0.001
		Ruminococcus_2	0.008	0.006	0.002
		Family_XIII_ge	0.008	0.006	0.001
		F082_ge	0.008	0.006	0.005
		Mogibacterium	0.007	0.005	0.001
		uncultured_ge	0.007	0.002	0.001
		Rikenellaceae_RC9_gut_group	0.007	0.004	0.008

		Muribaculaceae_ge	0.006	0.004	0.007
		Lachnospiraceae_UCG.008	0.006	0.002	0.001
		JGI_0000069.P22_ge	0.006	0.002	0.001
		Desulfovibrio	0.005	0.002	0.001
		Fretibacterium	0.004	0.003	0.001
		Family_XIII_AD3011_group	0.004	0.002	0.001
		Comamonas	0.004	0.002	0.001
		Succinivibrionaceae_UCG.002	0.004	0.003	0.013
		Anaerovorax	0.003	0.001	0.001
		CPla.4_termite_group	0.003	0.002	0.025
		Ruminococcaceae_ge	0.003	0.001	0.001
		Lachnospiraceae_UCG.010	0.002	0.001	0.001
		Lachnoclostridium_1	0.002	0.001	0.019
		Prevotellaceae_UCG.004	0.002	0.001	0.001
		U29.B03	0.002	0.001	0.001
		Oribacterium	0.002	0.001	0.005
RS x RE	High Starch	Christensenellaceae_R.7_group	0.088	0.019	0.001
		Lachnospiraceae_NK3A20_group	0.047	0.013	0.001
		Ruminococcaceae_NK4A214_group	0.024	0.007	0.001
		Butyrivibrio_2	0.022	0.010	0.001
		Desulfobulbus	0.020	0.009	0.001
		Campylobacter	0.018	0.015	0.001
		Prevotellaceae_UCG.001	0.014	0.005	0.001
		Treponema_2	0.014	0.017	0.001
		Succiniclasticum	0.014	0.006	0.001
		Acetitomaculum	0.012	0.010	0.001
		Howardella	0.011	0.006	0.001
		Family_XIII_ge	0.011	0.009	0.002
		uncultured	0.010	0.006	0.003
		Saccharofermentans	0.009	0.003	0.001
		Lachnospiraceae_UCG.008	0.007	0.004	0.001
		Muribaculaceae_ge	0.007	0.004	0.001
		Comamonas	0.006	0.004	0.001
		Moryella	0.006	0.001	0.001
		JGI_0000069.P22_ge	0.005	0.004	0.001
		Bifidobacterium	0.005	0.005	0.004
		uncultured_ge	0.005	0.001	0.001
		Prevotellaceae_UCG.003	0.004	0.003	0.001
		Fretibacterium	0.004	0.002	0.001

		Anaerovorax	0.003	0.003	0.001
		DNF00809	0.003	0.001	0.001
		U29.B03	0.003	0.002	0.001
		Fibrobacter	0.003	0.002	0.002
		Oribacterium	0.003	0.001	0.001
		Lachnospiraceae_FE2018_group	0.003	0.001	0.001
		Lachnoclostridium_1	0.002	0.001	0.001
		Prevotellaceae_UCG.004	0.002	0.001	0.001
		Lachnospiraceae_UCG.010	0.002	0.001	0.001
		Lachnospira	0.002	0.007	0.036
	Low-Starch	Christensenellaceae_R.7_group	0.064	0.019	0.001
		Butyrivibrio_2	0.025	0.009	0.001
		Desulfobulbus	0.023	0.008	0.001
		Ruminococcaceae_NK4A214_group	0.021	0.006	0.001
		Lachnospiraceae_NK3A20_group	0.020	0.019	0.005
		Campylobacter	0.018	0.008	0.001
		Succiniclasticum	0.018	0.005	0.001
		unclassified	0.015	0.009	0.001
		uncultured	0.011	0.004	0.001
		Lachnospiraceae_XPB1014_group	0.011	0.004	0.001
		Prevotellaceae_UCG.001	0.010	0.005	0.001
		Howardella	0.010	0.004	0.001
		Ruminococcus_2	0.009	0.005	0.001
		Saccharofermentans	0.008	0.002	0.001
		Family_XIII_ge	0.008	0.006	0.001
		Rikenellaceae_RC9_gut_group	0.008	0.005	0.001
		Ruminococcaceae_UCG.014	0.008	0.004	0.015
		F082_ge	0.007	0.004	0.029
		uncultured_ge	0.007	0.002	0.001
		Muribaculaceae_ge	0.007	0.004	0.001
		Mogibacterium	0.007	0.005	0.001
		Lachnospiraceae_UCG.008	0.006	0.002	0.001
		JGI_0000069.P22_ge	0.006	0.002	0.001
		Desulfovibrio	0.005	0.002	0.001
		Flexilinea	0.005	0.003	0.001
		Fretibacterium	0.004	0.003	0.001
		Comamonas	0.004	0.002	0.001
		Family_XIII_AD3011_group	0.004	0.002	0.001
		Succinivibrionaceae_UCG.002	0.004	0.002	0.007

		Moryella	0.003	0.002	0.001
		Anaerovorax	0.003	0.001	0.001
		Ruminococcaceae_ge	0.003	0.001	0.001
		CPla.4_termite_group	0.003	0.002	0.004
		Lachnoclostridium_1	0.003	0.002	0.001
		Lachnospiraceae_UCG.010	0.002	0.001	0.001
		Oribacterium	0.002	0.001	0.001
		WCHB1.41_ge	0.002	0.001	0.001
		U29.B03	0.002	0.001	0.001
